# Sleep as a mediator of the relationship between social class and health in higher education students

**DOI:** 10.1111/bjop.12645

**Published:** 2023-03-09

**Authors:** Romany McGuffog, Mark Rubin, Mark Boyes, Marie L. Caltabiano, James Collison, Geoff P. Lovell, Orla Muldoon, Stefania Paolini

**Affiliations:** ^1^ University of Newcastle Callaghan New South Wales Australia; ^2^ Durham University Durham UK; ^3^ Curtin University Perth Western Australia Australia; ^4^ James Cook University Douglas Queensland Australia; ^5^ Australian College of Applied Psychology Sydney New South Wales Australia; ^6^ The University of the Sunshine Coast Sippy Downs Queensland Australia; ^7^ University of Limerick Limerick Ireland

**Keywords:** mediation, mental health, physical health, sleep, social class

## Abstract

A substantial body of research indicates that higher education students from lower social class backgrounds tend to have poorer health than those from higher social class backgrounds. To investigate sleep as a potential mediator of this relationship, online survey responses of students from five large Australian universities, one Irish university and one large Australian technical college were analysed in three studies (Study 1 *N* = 628; Study 2 *N* = 376; Study 3 *N* = 446). The results revealed that sleep quality, sleep duration, sleep disturbances, pre‐sleep worries and sleep schedule variability mediated the relationship between social class and physical and mental health. Sleep remained a significant mediator when controlling for related variables and other mediators. Thus, the findings suggest that sleep partly explains social class differences in health. We discuss the importance of addressing sleep issues among students from lower social class backgrounds.

## BACKGROUND

Substantial research demonstrates that compared to people from higher social class backgrounds, people from lower social class backgrounds tend to have poorer mental and physical health (Euteneuer, 2014; Foverskov & Holm, 2016; Kagamimori et al., 2009; Kim et al., 2015). Importantly, this relationship between social class and health has been replicated across different age groups and countries (Bøe et al., 2012; Demakakos et al., 2008; Howell & Howell, 2008; Kagamimori et al., 2009; Kim et al., 2015; Rubin et al., 2016). Furthermore, Stringhini et al. (2017) found that being from a lower social class background reduced one's life expectancy by 2.1 years, which ranked social class as having the third highest impact on life expectancy, following smoking (4.8 years) and diabetes (3.9 years).

This health disparity is particularly important for university students, who often experience poorer health than the general population. For example, a study by Auerbach et al. (2018) found that university students from a number of countries (including Australia) reported higher numbers of mental health problems and/or disorders compared to the general population. Studies on Australian students also found that compared to matched age comparators, students reported significantly poorer mental and physical health (Lovell et al., 2015; Stallman, 2010). Furthermore, education is the key route to social mobility in meritocratic societies, which highlights that higher education is a particularly relevant context in which to study social class (Bathmaker et al., 2016). Recently, there has been an increase in the proportions of low socio‐economic status (SES) students at universities, and the Australian government has invested nearly $1.5 billion into getting more low SES into universities (O'Shea, 2022). understanding what drives these social class differences is therefore critical in order to reduce health inequalities and improve the well‐being of higher education students.

Various explanations for the relationship between social class and health have been proposed. For example, previous research has suggested that higher education students from lower social class backgrounds may experience poorer health because of less social support, less physical activity, less social contact and poorer diet quality (Adler et al., 2000; Chen & Miller, 2013; Rubin et al., 2016; Rubin & Kelly, 2015; Stephens et al., 2012). In the present research, we focused on sleep as a possible mediator of the relationship between social class and health. Sleep plays a key role in maintaining metabolic, endocrine and immune functioning (Bagley et al., 2015). Thus, sufficient and restorative sleep is critical for the maintenance of physical and mental health (Lund et al., 2010). The reported restorative role of sleep appears particularly relevant when noting that people from lower social class backgrounds tend to have poorer sleep (Etindele Sosso et al., 2022; Felden et al., 2015; Mezick et al., 2008). Differences in sleep environments (i.e. factors in an individual's sleeping location that can impact sleep, such as light, noise, temperature, etc.) are often cited as an explanation for this relation (e.g. Bagley et al., 2015). Research has also found that a high number of university students have less than the recommended sleep for young adults and poor sleep quality in both American students (Lund et al., 2010) and Australian students (Lovell et al., 2015).

### Previous research on social class, sleep and health

Previous research has shown that people who experience poorer sleep also tend to report poorer health (e.g. Furihata et al., 2012; Lund et al., 2010). Research has also shown that people from lower social class backgrounds tend to experience poorer sleep (e.g. Felden et al., 2015; Mezick et al., 2008), which some research has suggested is due to factors such as poorer sleep environments, longer work hours and more shift work (Papadopoulos et al., 2022). However, relatively few papers have explored the possibility that social class differences in health are related to social class differences in sleep duration and quality, particularly in university students. Preliminary research by Van Cauter and Spiegel (1999) and Adler et al. (2000) were among the first to propose that sleep could mediate the relationship between social class and health. Further research has attempted to test sleep as a mediator of this relation (Bøe et al., 2012; Green et al., 2014; Huynh & Chiang, 2016; Moore et al., 2002; Sekine et al., 2006; Soltani et al., 2012). However, this past work suffers from two key limitations regarding methodology and analysis.

First, researchers have tended to use limited measures of sleep, health and social class. For example, past studies generally only considered objective social class (such as education and income) or only mental health. In addition, sleep is made up of a number of aspects, and it is important to investigate each aspect as a potential mediator of the relationship between social class and health. For example, sleep disturbances may be a significant mediator, but sleep duration may not. By limiting research focus to broad constructs such as sleep quality, one cannot pinpoint the specific aspects of sleep that may be at play. Finally, mental health and physical health are different constructs, and it cannot be assumed that the mediating effect of sleep operates in the same way in relation to both. This limited approach would prevent researchers from understanding which particular aspects of sleep and health are involved in the putative mediation effects, and it would not allow for a fuller exploration of social class and all of the aspects involved in the concept of social class. To address these issues, we included a variety of different measures of social class, sleep and health in our studies of sleep and health, and we combined various items to create a global score of social class, following the approach outlined by Rubin and Wright (2015).

The second limitation is that researchers have used statistical analyses (such as ANCOVA or regressions) that do not test the significance of the putative mediation effect. By using these analysis methods, the researchers were able to control for sleep, but not test whether sleep acted as a significant mediator variable. In other words, researchers were able to demonstrate that the total effect of social class on health was significant and that this effect then became non‐significant when controlling for sleep (i.e. the direct effect). However, they were not able to test whether the difference between the total effect and the direct effect (i.e. the indirect effect) was significant. While the direct effect is useful to investigate, a scoping review of the mediating effect of sleep on the relationship between social class and health highlighted that more research is needed on the indirect effect (Papadopoulos et al., 2022).

There is growing evidence that resolving sleep problems can help to treat mental illness (Anderson, 2015). For example, the results of Gee et al.'s (2018) meta‐analysis indicated that non‐pharmacological sleep interventions (such as stimulus control therapy, relaxation training, sleep restriction therapy, sleep hygiene and multicomponent cognitive behavioural therapy for insomnia) could help to reduce depressive symptoms and potentially anxiety. Gee et al. highlighted that using non‐pharmacological sleep interventions can be cost‐effective and that these interventions are useful for people with depression who are not interested in getting treatment specifically for their depression. Thus, non‐pharmacological sleep interventions could be cheap and useful to help improve students' sleep and subsequently their mental health.

### The present research

We used three studies to investigate the mediating effect of sleep on the relationship between social class and health in higher education students. Based on previous research in this area, we focused on broadening the measures used for social class, sleep and health and using a more targeted analytical approach to test for putative mediation effects.

#### Approach to measuring key variables

Although social class and SES are often used interchangeably, they refer to different constructs (Côté, 2011; Ostrove & Cole, 2003). Social class can be seen as a more umbrella term that encompasses socio‐economic status (which can include objective markers such as income and education) and subjective social status (which can include perceptions of your social rank in relation to others; American Psychological Association, 2023; Côté, 2011; Deutsch, 2017; Rubin et al., 2014). However, it is important to note that due to the complexity of social class as a concept, it can be measured in a number of different ways (Côté, 2011) and definitions can differ (Côté, 2011).

Consequently, for the present research, we similarly decided to focus more globally on social class, which meant that we could measure elements of socio‐economic status (e.g. education level), and subjective social status (e.g. perceived social class). Using this global approach is beneficial for our target population of higher education students since most university students have not had a chance to establish their SES because they are not yet in the workforce (Evans et al., 2022). Evans et al. (2022) also highlight the usefulness of modifying some measures to refer to participants' parents. For example, asking university students to provide their highest level of education is likely to provide extremely similar results across all participants, however, asking participants to provide the highest level of education for their parents is likely to provide more diversity and tap into the sociocultural background of participants. Furthermore, we also treated social class as a continuous variable, following Rubin et al. (2019) and Evans et al. (2022). Treating social class as a continuous variable is useful because we were interested in exploring the continuous associations between social class and sleep and health rather than assessing mean differences between categories. Additionally, treating social class continuously accounts for variability within potential categories of social class.

Since sleep involves numerous aspects, we measured sleep quality as an overall measure of sleep, and we also measured several key aspects of sleep such as disturbances, duration, daytime sleepiness, pre‐sleep worries and sleep schedule variability. This approach allowed us to investigate whether there were particular aspects of sleep that played more of a role in the relationship between social class and health. In a similar vein, we measured various aspects of mental and physical health including physical health symptoms, general physical health, general distress and self‐esteem.

#### Hypotheses

We used three studies to explore two hypotheses. Study 1 aimed to recruit participants from varying universities around Australia. Study 2 provided a conceptual replication of Study 1 and also included additional covariates and prospective mediators to be explored. Study 2 was also recruited from a different Australian university and a technical college. Study 3 aimed to also explore sleep hygiene and recruit additional participants from an Irish university. Study 3 followed a pre‐registered design. For the full pre‐registration document, surveys and anonymized data files, see the Open Science Foundation (OSF) folder at https://osf.io/mj43c/?view_only=f8f1cf89bd244974aee577ee7d6be691. However, for the sake of brevity and consistency of reporting across the studies, we have simplified the wording of the hypotheses.

Hypothesis 1 was that sleep (sleep quality, daytime sleepiness, sleep duration, sleep disturbances, pre‐sleep worries and sleep schedule variability) partly explains the relationship between social class and health. This hypothesis was tested across all three studies. We tested the robustness of this mediation effect. First, we aimed to show that the mediating effect of sleep on the relationship between social class and health remained significant after controlling for a number of variables that, based on previous research, may be related to the three variables, such as age, ethnic minority status, gender, time of day of survey completion, eveningness, physical activity and diet quality. Second, we aimed to compare the different aspects of sleep directly to determine which aspect of sleep (e.g. quality, duration, disturbances, etc.) was driving the mediation effect and whether these variables operated as mediators independently or whether one of them accounted for the mediation effects of the other measures.

For Hypothesis 2 (which was tested in Study 3), we investigated whether social class impacted students' ability to improve their sleep. Thus, we aimed to explore the feasibility of a simple method to improve sleep: sleep hygiene. Sleep hygiene involves improving sleep habits through various strategies, including improving the sleep environment (Better Health Channel, 2014; Sleep Health Foundation, 2011). As students from lower social class backgrounds may have less opportunity to implement some of these sleep habits, Hypothesis 2 tested whether, compared to students from higher social class backgrounds, students from lower social class backgrounds would report that implementing sleep hygiene techniques would be more difficult. To test this hypothesis, we created a scale to measure the perceived feasibility of implementing various sleep hygiene techniques. Although this measure has some commonalities with the Sleep Hygiene Index (Mastin et al., 2006), our measure explores additional sleep hygiene techniques that are identified by various health/sleep organisations (e.g. Better Health Channel, Sleep Health Foundation). Our measure also focuses on the perceived feasibility of the techniques rather than whether students actually engage in these behaviours.

## METHOD

### Participants and design

To investigate the relationship between social class (predictor variable), sleep (mediator variable) and mental and physical health (outcome variables), a cross‐sectional correlational design was used in combination with quantitative, self‐report measures. The key analyses used in the research were correlation and mediation.

We used Schoemann et al.'s (2017) Monte Carlo power analysis for the indirect effects app (https://schoemanna.shinyapps.io/mc_power_med/) to determine the required sample size. To detect a mediation effect with correlations of .15 (predictor and mediator), .15 (predictor and outcome) and .29 (mediator and outcome), a power value of .85 would require 372 participants. Thus, the sample sizes of each study (Study 1 *N* = 628; Study 2 *N* = 376; Study 3 *N* = 446) had sufficient power.

We recruited participants from five large Australian universities, one Irish university, and one large Australian technical college. For the Australian universities, we aimed to recruit universities that had a large proportion of low SES in order to obtain a good representation of students from varying social class backgrounds. Following the National Centre for Student Equity in Higher Education (2015) data, the representation of low SES at each of these universities ranged from 13.98% to 29.63%. We chose to recruit from an Australian technical college because they also have a good representation of social class, due to its focus on vocation, which leads to a broad student population that is less likely to have upper‐class or middle‐class values (James, 2000). Technical colleges provide vocational education and training, with diplomas and certificates in a wide range of courses such as accounting, childcare, trades and beauty therapy (see the Supplementary Document Tables S4–S8 for a detailed breakdown of the frequencies for each social class item). In particular, as seen in Table S7, approximately 13% of participants self‐identified as working class across all three studies.

Table 1 provides a breakdown of participant demographics for each study, including sample sizes from each population, gender, age and ethnic minority status.

**TABLE 1:** Demographic variables for each study.

	Study 1, *N* = 628 (%)	Study 2, *N* = 376 (%)	Study 3, *N* = 446 (%)
Population numbers	University 1 (*n* = 212, 33.76%)	University 5 (*n* = 342, 90.96%)	University 5 (*n* = 380, 85.20%)
	University 2 (*n* = 110, 17.52%)	Technical College (*n* = 34, 9.04%)	University 6 (*n* = 66, 14.80%)
	University 3 (*n* = 113, 17.99%)		
	University 4 (*n* = 193, 30.73%)		
Gender			
Male	124 (19.75%)	71 (18.88%)	88 (19.73%)
Female	503 (80.10%)	304 (80.85%)	358 (80.27%)
Other	1 (0.16%)	1 (0.27%)	0 (0%)
Age			
*M*	23.28	22.93	23.00
*SD*	8.53	6.71	7.18
Ethnicity			
White	423 (67.36%)	318 (84.57%)	383 (85.87%)
Aboriginal	16 (2.55%)	10 (2.66%)	12 (2.69%)
Torres Strait Islander	0 (0%)	0 (0%)	1 (0.22%)
Asian	73 (11.62%)	22 (5.85%)	26 (5.83%)
African	15 (2.39%)	3 (0.80%)	4 (0.90%)
Mixed race^a^	0 (0%)	0 (0%)	2 (0.45%)
Irish Traveller^a^	0 (0%)	0 (0%)	0 (0%)
Other	97 (15.45%)	19 (5.05%)	16 (3.59%)

^a^ Ethnicity option that was only provided to the Irish sample.

### Procedure

All participants for each study completed an online survey. (For full versions of the surveys, please see the OSF Folder). University students from both Australia and Ireland were recruited through each university's online research participation system. They received course credit in exchange for taking part. The participants from the technical college were recruited through flyers, posters and social media. The technical college participants were entered into a prize draw to win 1 of 11 $150 electronic gift certificates, with at least a 1 in 18 chance of winning.

The surveys ranged from 20 to 40 min in length (Study 1 = 25 min; Study 2 = 30 min; Study 3 = 40 min). The presentation order of the sleep and health measures was randomized. Physical activity and diet quality were included in the same section as the health measures. The scales and items within each section were randomized except for the Pittsburgh Sleep Quality Index. Following Langhout et al. (2009), the measures of social class were placed near the end of the research questionnaire together with the demographic variables in order to avoid cuing participants to the relevance of social class prior to their completion of the outcome variables.

### Measures

#### Social class measures

##### Education level

As education level is the most widely used proxy for social class (e.g., Kraus & Stephens, 2012; Martin, 2012; Martinez et al., 2009), we used two items to assess the highest education levels of participants' parents. The categories used for education levels were based on the education level items in Rubin and Wright's (2015) study. An example of an item is ‘the highest education level achieved by my mother was/is’, with responses on an 8‐point scale ranging from *less than primary school* (1) to *University or College of Advanced Education – postgraduate degree (Masters or PhD)* (8).

##### Occupational prestige and status

Following Rubin and Wright (2015), we also included two items that assessed participants' beliefs about the prestige and status of each of their parents' occupations. An example item is ‘Please indicate how you think most people would rate the prestige and status of the main occupation of your MOTHER. If your mother is or was mainly “unemployed” or a “homemaker,” then please count this as her occupation and rate its prestige/status. If you are not sure about the answer to this question, then please make your best guess’. Responses were made on an 11‐point scale ranging from *extremely low status and prestige* (0) to *extremely high status and prestige* (10).

##### Childhood socio‐economic status

We also included three items that assessed childhood socio‐economic status. These items were based on those used by Griskevicius et al. (2011). An example item is ‘my family usually had enough money to buy things when I was growing up’. Participants responded on a 7‐point scale ranging from *strongly disagree* (1) to *strongly agree* (7).

##### Subjective social class

There were also three items that assessed the subjective social class of the participant and their parents (Rubin et al., 2014). The participants responded to items such as ‘my father's social class was/is’ using a 5‐point scale ranging from *working class* (1) to *upper‐class* (5).

##### MacArthur Subjective Social Status scale

Finally, we included a modified version of the MacArthur Subjective Social Status scale (Adler et al., 2000). The scale contained one item: ‘Below, you will see a scale of 11 levels ranging from the top level to the bottom level. Please think of this scale as levels that represent where people stand in society. At the top levels are the people who are best off – those who have the most money, the most education, and the most respected jobs. At the bottom levels are the people who are worse off – who have the least money, the least education, and the least respected jobs or no job. The higher your level, the closer you are to the people at the very top; the lower you are, the closer you are to the people at the very bottom. Thinking about your current situation, please indicate where you would place yourself on this scale relative to other people in Australia/Ireland’. The scale used an 11‐point scale that was anchored *bottom level* (1) and *top level* (11).

All of the social class items mentioned in this section were used across all three studies. Following previous research (e.g. Rubin & Wright, 2015), we computed an overall global index of social class by converting the scores for each item into *z* scores (αs from the current studies ranged from .84 to .86). The inter‐correlations between each item was significant and ranged from .08 to .59. For a breakdown of the inter‐correlations between the individual social class items, see Tables S9–S11 in the Supplementary Document. The global score of social class was scored in such a way that a high score refers to a higher social class.

#### Sleep measures

The sleep measures were scored so that higher scores indicated poorer sleep. The sleep measures were present in all three studies.

##### Sleep quality

Our measure of sleep quality included 18 items from the Pittsburgh Sleep Quality Index (Buysse et al., 1989). This measure assesses seven areas of sleep in the last month: subjective sleep quality, sleep latency, sleep duration, habitual sleep efficiency, sleep disturbances, use of sleep medication and daytime dysfunction (Buysse et al., 1989). Following the scoring provided by Buysse et al., we used this measure as a global score (αs from the current studies ranged from .64 to .68). The response scales for this measure vary for each item. An example item is ‘During the past month, when have you usually gone to bed?’

##### Sleep duration

The measure for sleep duration differed between studies. In Studies 1 and 2, we used the sleep duration subscale of the Pittsburgh Sleep Quality Index. In Study 3, we included an additional single item based on the sleep duration item in the Medical Outcomes Study Sleep Scale (Hays et al., 2005). This item was ‘On average, how many hours did you sleep each night during the past 4 weeks?’ and had an open‐ended response. To be consistent with the scoring of the other sleep measures, we coded the sleep duration item so that a higher score indicated shorter sleep.

##### Sleep disturbances

We used a 22‐item Sleep Disturbance Questionnaire that was developed in order to provide a more detailed measure of the specific reasons for sleep disturbance (McGuffog & Rubin, 2015; for additional details on the development of the scale and psychometric properties, see Tables S1–S3 in the Supplementary Document). Participants were asked to rate the frequency of eight different disturbances (stress/anxiety/worry, climate, stimuli, activities, poor sleep routine, pain/discomfort, bodily needs and dreams/nightmares) across three stages of sleep (falling asleep, during sleep and waking up). The response scale ranged from *never* (1) to *all the time* (7). An example item was ‘Over the past four weeks, how often does each of the following make it difficult for you to fall asleep at night?’, and an example disturbance included ‘Stimuli inside or outside of your room (e.g. noises, light, other people, pets, animals, birds, etc.)’.

##### Daytime sleepiness

We also used the Stanford Sleepiness Scale (Hoddes et al., 1973) to assess participants' current level of sleepiness as they completed the survey. This scale contained one item: ‘please indicate how sleepy you feel right now’. Participants responded on a scale ranging from *feeling active, vital, alert or wide awake* (1) to *no longer fighting sleep, sleep onset soon; having dream‐like thoughts* (7).

##### Pre‐sleep worries

We adapted Bagley et al.'s (2015) measure of pre‐sleep worries to be appropriate for an adult population. This measure included five items about worries related to friends, family, university, work, and other issues. An example item includes ‘How often does worrying about your friends affect your sleep?’ Participants responded on a scale that ranged from *never* (1) to *all the time* (7). We treated this measure as a global score (αs from the current studies ranged from .81 to .84).

##### Sleep schedule variability

Finally, we used a single item to measure sleep schedule variability, based on Duncan et al. (2015): ‘On average, how regular are you in going to bed and getting up at the same times each day (including weekends)?’ Responses ranged from *extremely irregular* (1) to *extremely regular* (6). To be consistent with the scoring of the other sleep measures, we coded this item so that a higher score indicated more variability.

#### Mental and physical health measures

##### General physical health

To measure physical health, we used Ware's (1976) 10‐item Health Perceptions Questionnaire (αs from the current studies ranged from .88 to .90). An example item is ‘I am somewhat ill.’ To maintain consistent wording with many of our other measures that use response scales based on level of agreement, we adapted the response scale from a 5‐point scale to a 7‐point scale ranging from *strongly disagree* (1) to *strongly agree* (7).

##### Physical health symptoms

We also included the 14‐item Physical Health Questionnaire (Schat et al., 2005). This scale measures common somatic symptoms including gastrointestinal problems, headaches, sleep disturbances and respiratory infections. An example item of the scale is ‘How often have you experienced headaches?’ We removed the four sleep items because we were already measuring sleep using various other measures. Participants responded on a 7‐point scale ranging from *never* (1) to *all the time* (7). We used this measure as a global score of physical health symptoms (αs from the current studies ranged from .84 to .90).

##### General distress

To measure mental health problems, we used the 21‐item version of the Depression Anxiety Stress Scale (Lovibond & Lovibond, 1995). The scale requires participants to consider how they have felt during the past week; it is split into three subscales: depression, anxiety and stress. Some example items include ‘I felt down‐hearted and blue’ (from the depression subscale), ‘I found it difficult to relax’ (from the stress subscale) and ‘I felt I was close to panic’ (from the anxiety subscale). Participants responded using a 4‐point scale, ranging from *never* (0) to *almost always* (3). Following Zanon et al. (2020), we decided to use this scale as a global measure of general distress^1^ (αs = .94 across all three studies).

##### Self‐esteem

Finally, we measured self‐esteem using a single item (Robins et al., 2001): ‘I have high self‐esteem’. The measure used a 7‐point response scale ranging from *strongly disagree* (1) to *strongly agree* (7). The health measures were scored so that a higher score indicates poorer mental and physical health, except for self‐esteem, in which a higher score refers to higher self‐esteem.

#### Control measures

We measured several variables that had the potential to impact social class, sleep and/or health, including age, ethnic minority status, gender, time of day of survey completion, eveningness, physical activity and diet quality. First, age is important to consider because a study by Rubin and Wright (2015) found that age and social class were negatively related, indicating that in university, the higher the social class, the younger the person.

Controlling for ethnic minority status is also important because there is evidence to show that social class proportions can differ in different ethnicities, with minority ethnicities such as African Americans in the United States reporting being in lower social classes than individuals from majority groups such as white individuals (Mezick et al., 2008). In fact, there are even some studies that include race/ethnicity as a measure of SES (e.g. Stamatakis et al., 2007). So, for our measure of ethnic minority status, the response options included ‘Caucasian’, ‘Asian’, ‘African’, ‘Aboriginal’, ‘Torres Strait Islander’ and ‘other’. These response options were then coded into Caucasian versus non‐Caucasian to identify ethnic minority status.

Gender was also measured because previous research has found gender differences in sleep such that women reported earlier bed and rise times, longer sleep latency, more sleep disturbances, poorer sleep quality and were more likely to develop insomnia (Green et al., 2014; Ozrech et al., 2011; Tsai & Li, 2004).

In addition, participants were also asked to record the time of day that they completed the survey (morning, afternoon/evening, night) in order to control for this variable in our analyses. The circadian rhythm of the body means that attention and sleepiness can wax and wane at different times of the day (Price, 2011). Thus, it is important to measure the time of day of survey completion to control in our analyses, particularly when considering daytime sleepiness.

Additionally, research has demonstrated that being an evening‐active person is associated with poorer sleep and health in students/young adults (e.g. Jankowski, 2016; Tavernier & Willoughby, 2014). Thus, we included a measure of eveningness based on Vink et al. (2001), to control for any impact being a morning person versus an evening person could have on one's sleep and health variables. This measure contained a single item: ‘Are you a morning‐active person or an evening‐active person?’ Responses ranged from *morning‐active* (1) to *evening‐active* (5).

Less physical activity has been found to be linked to lower social class indicators (e.g. Bauman et al., 2012) and poorer sleep (e.g. Kredlow et al., 2015). Thus, we included a single item by Milton et al. (2010): ‘In the past week, on how many days have you done a total of 30 minutes or more of physical activity, which was enough to raise your breathing rate (this may include sport, exercise, and brisk walking or cycling for recreation or to get to and from places, but should not include housework or physical activity that may be part of your job)?’ This item has a response scale ranging from 0 days to 7 days.

Finally, previous research has also highlighted that low SES is associated with poorer diet quality (e.g. Darmon & Drewnowski, 2008). Therefore, to measure diet quality, we asked participants to rate the average healthiness of their diet over the past 4 weeks, ranging from *extremely unhealthy* (1) to *extremely healthy* (9).

#### Perceived feasibility of sleep hygiene techniques

We developed a new measure for Study 3 to assess the perceived feasibility of sleep hygiene techniques. Organizations such as Better Health Channel (2014) and Sleep Health Foundation (2011) recommend several strategies for improving sleep habits. We adapted these proposed strategies into a 21‐item measure and asked participants to consider how easy it would be for them to follow each strategy to help improve their sleep. Some examples included ‘not ignoring tiredness and going to bed when you feel ready’, ‘having a bed that is comfortable’ and ‘making your sleep environment quiet’. Participants responded using a 6‐point response scale ranging from *extremely difficult for me to do* (1) to *extremely easy for me to do* (6).

## RESULTS

Please note that although we undertook multiple significance tests in our studies, we did not undertake multiple tests of the *same joint null hypothesis* and reject that joint null hypothesis following *at least one* significant result (*disjunction testing*; Rubin, 2021). Instead, we undertook single tests of multiple *individual* null hypotheses (*individual testing*; Rubin, 2021). Following Rubin (2021), an alpha adjustment is only appropriate in the former case and not in the latter case.

Hypothesis 1 : The mediating effect of sleep on the relation between social class and health.

Preliminary analyses were conducted before performing the mediation tests. Normality was checked and, when necessary, addressed (please see the Supplementary Document for details). The correlation analyses indicated the key aspects of sleep and health that would be included in the mediation tests (see Tables 2, 3, and 4). In particular, it is possible that (a) sleep quality, (b) sleep disturbances (Studies 1 and 3 only), (c) sleep duration, (d) pre‐sleep worries and (e) sleep schedule variability (Studies 1 and 2 only) mediate the relationship between social class and physical health symptoms, general physical health, general distress and self‐esteem.

**TABLE 2:** Social class and mental and physical health: means, standard deviations, cronbach alphas and correlations with social class.

	Study 1			Study 2			Study 3		
	*M* (*SD*)	Cronbach alpha	Correlation with social class	*M* (*SD*)	Cronbach alpha	Correlation with social class	*M* (*SD*)	Cronbach alpha	Correlation with social class
Social class	0.00 (0.56)	0.84	–	0.00 (0.64)	0.86	–	0.00 (0.64)	0.86	–
Physical health symptoms	2.97 (1.12)	0.87	−.11**	3.10 (1.13)	0.86	−.13**	3.03 (1.05)	0.84	−.18**
General physical health	3.94 (1.10)	0.88	−.25**	4.01 (1.21)	0.90	−.22**	3.77 (1.15)	0.89	−.19**
General distress	1.88 (0.57)	0.94	−.17**	1.83 (0.57)	0.94	−.17**	1.85 (0.58)	0.94	−.23**
Self‐esteem	4.09 (1.65)	–	.16**	3.83 (1.74)	–	.16**	4.13 (1.67)	–	.17**

*Note*: The response scales for physical health symptoms, general physical health and self‐esteem ranged from 1 to 7. The response scales for general distress ranged from 0 to 4. The social class items were converted to *z* scores and then averaged to form a single index.

** *p* < .01.

**TABLE 3:** Social class and sleep: means, standard deviations, cronbach alphas, and correlations with social class.

	Study 1			Study 2			Study 3		
	M (*SD*)	Cronbach alpha	Correlation with social class	M (*SD*)	Cronbach alpha	Correlation with social class	M (*SD*)	Cronbach alpha	Correlation with social class
Sleep quality	1.15 (0.47)	0.66	−.16**	1.20 (0.48)	0.64	−.17**	1.16 (0.47)	0.68	−.22**
Daytime sleepiness	3.22 (1.25)	–	−.07	3.36 (1.22)	–	−.08	3.36 (1.22)	–	−.07
Sleep disturbances	3.04 (1.01)	0.92	−.08*	3.10 (1.03)	0.92	−.09	3.32 (0.97)	0.92	−.16**
Sleep duration	1.02 (0.82)	–	−.18**	0.95 (0.81)	–	−.13**	16.88 (1.26)	–	−.11*
Pre‐sleep worries	3.31 (1.30)	0.84	−.12**	3.34 (1.25)	0.83	−.11*	3.65 (1.19)	0.81	−.15**
Sleep schedule variability	3.54 (1.32)	–	−.10*	3.70 (1.31)	–	−.11*	3.79 (1.35)	–	−.01

*Note*: The response scales for daytimes sleepiness, sleep disturbances and pre‐sleep worries ranged from 1 to 7. The response scale for sleep quality ranged from 0 to 3. The response scale for sleep schedule variability ranged from 1 to 6. The response scale for sleep duration in Studies 1 and 2 ranged from 0 to 4, and Study 3 ranged from 0 to 24.

*p < .05; **p < .01.

**TABLE 4:** Correlations between sleep measures and mental and physical health.

	Sleep quality	Daytime sleepiness	Sleep disturbances	Sleep duration^a^	Pre‐sleep worries	Sleep schedule variability
Study 1						
Physical health symptoms	.43**	.33**	.56**	.17**	.51**	.11**
General physical health	.47**	.32**	.42**	.26**	.42**	.22**
General distress	.59**	.35**	.58**	.24**	.62**	.17**
Self‐esteem	−.40**	−.29**	−.31**	−.17**	−.36**	−.18**
Study 2						
Physical health symptoms	.40**	.31**	.54**	.19**	.51**	.14**
General physical health	.37**	.34**	.36**	.14**	.33**	.25**
General distress	.60**	.34**	.53**	.28**	.62**	.27**
Self‐esteem	−.34**	−.20**	−.31**	−.10	−.37**	−.17**
Study 3						
Physical health symptoms	.41**	.26**	.51**	.11*	.47**	.13**
General physical health	.43**	.27**	.36**	.18**	.38**	.24**
General distress	.58**	.33**	.55**	.22**	.54**	.21**
Self‐esteem	−.42**	−.22**	−.37**	−.11*	−.41**	−.20**

^a^ Sleep duration measure in Studies 1 and 2 was a subscale within the sleep quality measure. In Study 3, the sleep duration measure was a separate item.

*p < .05; **p < .01.

**FIGURE 1:**
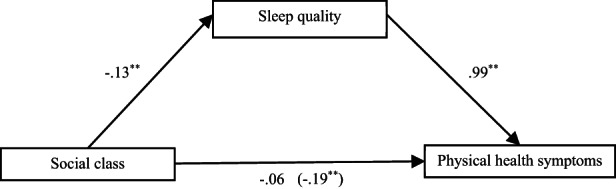
Mediation model showing the mediating effect of sleep quality on the relationship between social class and general physical health in Study 1 (see Table 5 for further statistical details for this model). Beta values are unstandardised. The value in parentheses represent total effect of social class on physical health symptoms in the absence of the mediator. **p < .01.

To test the potential mediation effects of sleep on the relationship between social class and health, we used Model 4 of Hayes' (2018) PROCESS software with 5000 iterations of bootstrapping. The results of mediation analyses are presented in Tables 5, 6, 7 (Tables S12–S21 in the Supplementary Document). We initially included each mediator in separate tests. We explain the first mediation test in detail. Thereafter, we summarize the results. The first test investigated sleep quality as a potential mediator of the relationship between social class and physical health symptoms. The total effect of social class on physical health symptoms was significant (*b* = −.19, *SE* = 0.07, *p* = .009, 95% CI [−0.33, −0.05]), the direct effect controlling for sleep quality was non‐significant (*b* = −.06, *SE* = 0.07, *p* = .342, 95% CI [−0.19, −0.07]), and the indirect effect was significant (*b* = −.12, *SE* = 0.03, 95% CI [−0.19, −0.07]). This pattern of results indicates a significant mediation effect, which is illustrated in Figure 1. The completely standardized indirect effect sizes (CSIES; Preacher & Kelley, 2011) was 0.07. When considering mediation effects, a small effect = 0.01, a medium effect = 0.09 and a large effect = 0.25 (Kenny, 2014). Hence, in this case, the indirect effect was medium in size. A slightly smaller significant reverse mediation effect (CSIES = 0.05) was also found, indicating that physical health symptoms mediated the relationship between social class and sleep quality (see Table 5).

**TABLE 5:** The mediating effect of sleep quality on the relationship between social class and each outcome variable (Study 1).

Outcome variables	Effect type	*b* (*SE*)	95% CI	*t*	*p*	CSIES (reverse)
Physical health symptoms	Total	−.19 (.07)	−0.33, −0.05	−2.61	.009	
	Direct	−.06 (.07)	−0.19, 0.07	−0.95	.342	
	Indirect	−.12 (.03)	−0.19, −0.07	–	–	−.07 (−.04)
General physical health	Total	−.44 (.07)	−0.57, −0.30	−6.38	<.001	
	Direct	−.31 (.06)	−0.43, −0.19	−4.98	<.001	
	Indirect	−.13 (.03)	−0.19, −0.07	–	–	−.07 (−.11)
General distress	Total	−.16 (.04)	−0.23, −0.08	−4.25	<.001	
	Direct	−.07 (.03)	−0.13, 0.01	−2.23	.026	
	Indirect	−.09 (.02)	−0.13, −0.05	–	–	−.09 (−.10)
Self‐esteem	Total	.49 (.10)	0.28, 0.69	4.64	<.001	
	Direct	.32 (.10)	0.13, 0.52	3.27	.001	
	Indirect	.16 (.04)	0.09, 0.25	–	–	.06 (−.07)

*Note*: Sleep quality is the mediator variable for all models. All models have Dfs of 2, 625. *SE* = standard error. 95% CIs = the upper and lower 95% confidence intervals; *SE*s and CIs for indirect effects are bootstrapped. If CIs are both positive or negative, then the indirect effect is significant at *p* < .05. The completely standardized indirect effect size (CSIES) for the reverse mediation effect is presented in parentheses after the original CSIES.

**TABLE 6:** The mediating effect of sleep quality on the relationship between social class and each outcome variable (Study 2).

Outcome variables	Effect type	*b* (*SE*)	95% CI	*t*	*p*	CSIES (reverse)
Physical health symptoms	Total	−.23 (.09)	−0.41, −0.06	−2.58	.010	
	Direct	−.11 (.08)	−0.28, 0.05	−1.35	.177	
	Indirect	−.12 (.04)	−0.19, −0.05	–	–	−.07 (−.05)
General physical health	Total	−.40 (.09)	−0.59, −0.22	−4.26	<.001	
	Direct	−.29 (.09)	−0.47, −0.12	−3.24	.001	
	Indirect	−.11 (.04)	−0.19, −0.05	–	–	−.06 (−.08)
General distress	Total	−.15 (.05)	−0.24, −0.06	−3.37	<.001	
	Direct	−.06 (.04)	−0.14, −0.01	−1.70	.091	
	Indirect	−.09 (.03)	−0.14, −0.04	–	–	−.10 (−.10)
Self‐esteem	Total	.44 (.14)	0.17, 0.71	3.20	.002	
	Direct	.29 (.13)	0.03, 0.55	2.20	.029	
	Indirect	.15 (.05)	0.06, 0.26	–	–	.06 (−.05)

*Note:* Sleep quality is the mediator variable for all models. All models have Dfs of 2, 373. See Table 1 note for further details.

**TABLE 7:** The mediating effect of sleep quality on the relationship between social class and each outcome variable (Study 3).

Outcome variables	Effect type	*b* (*SE*)	95% CI	*t*	*p*	CSIES (Reverse)
Physical health symptoms	Total	−.30 (.08)	−0.45, −0.15	−3.87	<.001	
	Direct	−.16 (.07)	−0.30, 0.02	−2.19	.029	
	Indirect	−.14 (.03)	−0.21, −0.08	–	–	−.08 (−.07)
General physical health	Total	−.33 (.08)	−0.50, −0.17	−3.97	<.001	
	Direct	−.17 (.08)	−0.33, −0.02	−2.18	.030	
	Indirect	−.16 (.04)	−0.24, −0.09	–	–	−.09 (−.07)
General distress	Total	−.21 (.04)	−0.29, −0.13	−5.01	<.001	
	Direct	−.10 (.04)	−0.17, −0.03	−2.81	.005	
	Indirect	−.11 (.03)	−0.16, −0.06	–	–	−.12 (−.13)
Self‐esteem	Total	.46 (.12)	0.22, 0.69	3.73	<.001	
	Direct	.23 (.12)	0.003, 0.45	1.99	.048	
	Indirect	.23 (.05)	0.12, 0.34	–	–	.09 (−.07)

*Note*: Sleep quality is the mediator variable for all models. All Models have Dfs of 2, 443. See Table 1 note for further details.

All other mediation tests were significant,^1^ except for three tests (sleep schedule variability as the mediator of the relationship between social class and general distress in Study 2; sleep duration as the mediator of the relationship between social class and physical health problems in Study 3 and sleep duration as the mediator of the relationship between social class and self‐esteem in Study 3). The other mediation tests also had significant reverse mediations that were similar in size to the original mediation effects. Thus, consistent with Hypothesis 1, the relationship between social class and health was in part explained by sleep. In this article, we chose to include only the tables showing sleep quality as the mediator across all three studies because sleep quality was our overall measure of sleep and it includes multiple aspects of sleep (see Tables 5, 6, 7 for details of the mediating effect of sleep quality across Studies 1, 2 and 3 and also see Tables S12–S21 in the Supplementary Document for the mediation results of sleep disturbances, sleep duration, pre‐sleep worries and sleep schedule variability).

### Testing the robustness of the mediating effect of sleep

#### Controlling for covariates

To further test the robust nature of the mediating effect of sleep on the relationship between social class and health, we reconducted the above mediation tests and included the control variables (age, ethnic minority status, gender, time of day of survey completion, eveningness, physical activity and diet quality as covariates). All 12 mediation results involving sleep quality as the mediator (four mediation models in each study) remained statistically significant even when including the covariates. When sleep disturbances were the mediator, four of the eight mediation results (four mediation models in both Studies 1 and 3) remained statistically significant. For sleep duration as the mediator, four of the 11 mediation results (four mediation models in both Studies 1 and 3, and three mediation models in Study 2) remained statistically significant. When pre‐sleep worries were entered as the mediator, eight of the 12 mediation results (four mediation models in each study) remained statistically significant. Finally, when sleep schedule variability was the mediator, five of the eight mediation results (four mediation models in both Studies 1 and 2) remained statistically significant. These results highlighted that sleep mostly persisted as a mediator of the relationship between social class and health, supporting Hypothesis 1.

#### Comparison between sleep mediators

To explore sleep as a mediator in more detail, we conducted parallel mediation tests in which we entered all sleep components (excluding sleep quality) as simultaneous mediators using Model 4. By entering the components of sleep simultaneously, we could examine which components of sleep had stronger effect sizes and were thus more relevant and useful in the relationship between social class and health. Across all three studies, pre‐sleep worries remained significant when compared to sleep disturbances, sleep duration, and sleep schedule variability. In contrast, sleep disturbances, sleep duration, and sleep schedule variables were often not significant, suggesting that there was some shared variance between the indirect effects that involved these sleep measures and pre‐sleep worries. For detailed results, see Tables S22–S24 in the Supplementary Document.

Hypothesis 2 : The mediating effect of sleep hygiene on the relation between social class and sleep.

In Study 3, we investigated the perceived difficulty of implementing sleep hygiene techniques. We considered three factors of the perceived feasibility of the sleep hygiene measure, including promoting good sleep, sleep environment and avoiding negative behaviours. However, due to the low internal reliability of the avoiding negative behaviours subscale (Spearman‐Brown split‐half coefficient = .50), we decided to investigate only correlations between social class and the other two measures. We found that students from lower social class backgrounds were more likely to report that improving their sleep environment would be more difficult (*r* = .12, *p* = .009). However, we did not find a significant relationship between social class and the promoting good sleep subscale (*r* = .06, *p* = .241). We also found that participants who reported that implementing behaviours that promote good sleep were less feasible also reported poorer sleep quality, more sleep disturbances, shorter sleep duration and more pre‐sleep worries (*r*s ≥ −.15, *p*s ≤ .001). This pattern of results was also found for students who reported that improving their sleep environment was less feasible (*r*s ≥ −.15, *p*s ≤ .001). For more details, see Tables S25 and S26 in the Supplementary Document for the correlation results.

In our non‐pre‐registered, exploratory analyses, we considered that the perceived difficulty of changing one's sleep environment may mediate the relationship between social class and sleep. We chose to only focus on sleep environment because it was the only subscale that correlated with both social class and sleep. We also only focused on the sleep variables that were correlated with social class. We found that the perceived difficulty of changing sleep environment mediated the relationship between social class and sleep quality, sleep duration, sleep disturbances, and pre‐sleep worries. Table 8 presents detailed information about the mediation effects. We also tested a serial mediation of social class → perceived feasibility of sleep environment hygiene → sleep → health which showed that the mediating effect of sleep hygiene was still significant when extending the mediation to also include health (see the Supplementary Document for more details).

**TABLE 8:** The mediating effect of perceived feasibility of sleep environment hygiene techniques on the relationship between social class and sleep measures (Study 3).

Outcome variables	Effect type	*b* (*SE*)	95% CI	*t*	*p*	CSIES
Sleep quality	Total	−.16 (.03)	−0.23, −0.09	−4.72	<.001	
	Direct	−.14 (.03)	−0.20, −0.07	−4.10	<.001	
	Indirect	−.03 (.01)	−0.05, −0.01	–	–	−.04
Sleep disturbances	Total	−.25 (.07)	−0.39, −0.11	−3.51	.001	
	Direct	−.19 (.07)	−0.32, −0.06	−2.78	.006	
	Indirect	−.06 (.03)	−0.11, −0.02	–	–	−.04
Sleep duration	Total	−.23 (.09)	−0.41, −0.04	−2.43	.016	
	Direct	−.19 (.09)	−0.37, −0.01	−2.04	.042	
	Indirect	−.04 (.02)	−0.08, −0.01	–	–	−.02
Pre‐sleep worries	Total	−.29 (.09)	−0.46, −0.12	−3.28	.001	
	Direct	−.23 (.09)	−0.40, −0.06	−2.71	.007	
	Indirect	−.05 (.02)	−0.10, −0.01	–	–	−.03

*Note*: Perceived feasibility of sleep environment hygiene techniques is the mediator variable for all models. See Table 1 note for further details.

## DISCUSSION

Previous research has found that (a) social class is related to mental and physical health (e.g. Foverskov & Holm, 2016), (b) sleep is related to mental and physical health (e.g. Lund et al., 2010) and (c) social class is related to sleep (e.g. Bagley et al., 2015). Although there is some research to suggest that sleep mediates the relationship between social class and health (e.g. Moore et al., 2002), there is limited research on higher education students. Additionally, these studies often have limited measures of social class, sleep and health, and they have failed to provide direct tests of mediation. In the current research, we explored this mediation effect using multiple measures of social class, sleep and health, and we tested the robustness of this mediation effect by controlling for covariates and comparing the sleep variables. Additionally, we explored the perceived difficulty of sleep hygiene techniques as a function of social class.

### The mediating effect of sleep on the relationship between social class and health

Before conducting the mediation analyses, we tested the correlations between social class, sleep and health. Across all three studies, we found that social class was related to a number of aspects of sleep (sleep quality, sleep duration, sleep disturbances, pre‐sleep worries and sleep schedule variability) and both mental health (general distress and self‐esteem) and physical health (physical health symptoms and general physical health). In contrast, we consistently found that social class was not significantly correlated with daytime sleepiness. It is possible that the single‐item measure of the current level of daytime sleepiness is not the most suitable way to measure this construct. Other research that has found a relationship between social class and daytime sleepiness has tended to measure average sleepiness using multi‐item scales (e.g. Bagley et al., 2015; Felden et al., 2015; Mezick et al., 2008). It is also possible that we did not find a relationship because self‐reports of sleepiness are not particularly accurate, as highlighted by Gawron (2016).

In terms of the mediation analyses, across all three studies, we found that sleep (sleep quality, sleep duration, sleep disturbances, pre‐sleep worries and sleep schedule variability) mediated the relationship between social class and both mental health (general distress and self‐esteem) and physical health (physical health symptoms and general physical health). There were only three tests that did not result in a significant mediation result. The mediation results suggest that sleep partly explains the relationship between social class and health, meaning that students from lower social class backgrounds are more likely to have poorer mental and physical health partly due to having poorer sleep. These findings suggest that sleep is an important factor to consider when exploring the relationship between social class and health.

To demonstrate the robustness of this mediation test, we conducted the mediation tests (a) controlling for covariates and (b) with all sleep mediators to determine the key sleep measures. Importantly, the mediating effect of sleep quality on the relationship between social class and health was not substantively impacted by the various covariates that we included. However, the mediation results including sleep disturbances, sleep duration, pre‐sleep worries and sleep schedule variability did not always remain significant when controlling for the covariates. These results highlight that some covariates may have shared some overlap with social class, sleep and/or health. Across all three studies, we found that pre‐sleep worries mediated the relationship between social class and health independent of other aspects of sleep including sleep disturbances, sleep duration and sleep schedule variability. The independence of pre‐sleep worries highlights the importance of focusing on factors that impact falling asleep.

### The impact of social class on students' ability to improve their sleep

Finally, in Study 3, we investigated the perceived difficulty of implementing sleep hygiene techniques. Consistent with our second hypothesis, we found that social class was negatively related to the perceived difficulty of changing one's sleep environment. Contrary to this hypothesis, social class was not associated with the promoting good sleep subscale. This pattern of results makes sense because difficulties in changing one's sleep environment are more likely to be affected by education levels and financial factors that are associated with social class. In particular, the sleep environment items were about removing unnecessary lights, and noises, having a good temperature and a comfortable bed. Thus, these techniques might be more difficult for participants from lower social class backgrounds than those suggested in the promoting sleep subscale, which included items about getting plenty of morning sunshine and using relaxation techniques to prepare for bed.

In our exploratory analyses, we investigated whether the perceived feasibility of sleep hygiene techniques mediated the relationship between social class and sleep. The mediation tests were consistent with a model in which students from lower social class backgrounds reported poorer sleep quality, more sleep disturbances, shorter sleep duration, and more pre‐sleep worries in part because they perceived implementing sleep environment hygiene techniques to be less feasible. It is possible that these effects only reflect attitudes about sleep hygiene rather than behaviours. However, from a theoretical perspective, it is unlikely that students have poorer sleep because they *perceive* sleep hygiene to be more difficult. It is more likely that perceiving sleep hygiene to be more difficult reflects the actual difficulty in implementing sleep hygiene techniques, which then impacts actual sleep quality.

### Limitations and future research

The cross‐sectional nature of the present research design does not allow us to draw clear conclusions about the causal directions of the relations between social class, sleep and health. There are some researchers who do not agree with using mediation analysis on cross‐sectional data (e.g. Maxwell & Cole, 2007). Although it is true that using cross‐sectional data is a limitation, this approach is not totally uninformative. For example, the absence of a significant mediation effect of sleep in the relationship between social class and health would represent a lack of evidence for the causal hypothesis that social class causes a lack of sleep, which causes poorer mental health. However, we did find consistent and robust mediation effects of sleep, which provides tentative evidence consistent with sleep mediating the relationship between social class and health. Thus, it remains important to demonstrate a mediation effect, even with cross‐sectional data, which then needs to be explored further for causation. Furthermore, past research has provided strong evidence that social class impacts sleep and health (Adler & Ostrove, 1999; Pope & Arthur, 2009). However, past research also suggests that the relationship between sleep and health is bidirectional (Kaneita et al., 2009). Consistent with this bidirectional relationship, our reverse mediations indicated that mental and physical health mediated the relationship between social class and sleep. Therefore, future research is required to investigate how social class, sleep and health are causally linked.^2^

It is also important that future research expands the exploration of both the sample and measures used. For example, it would be useful to complement the findings from the current research by utilizing objective sleep measures such as polysomnography or actigraphy, as recent reviews have highlighted the usefulness of these approaches in exploring the relationship between social class and sleep (Etindele Sosso, 2022; Etindele Sosso et al., 2021). Furthermore, our focus on university students limits the generalizability of our results to the general population. This is particularly evident in that the link between social class and university attendance (Rubin et al., 2019) means that our samples likely excluded the most disadvantaged end of the social class gradient. Moreover, our proportions of gender and ethnicity were somewhat skewed (mostly white and female), which highlights the importance of future research to recruit more gender‐balanced (including genders beyond male and female categories), and ethnically diverse samples. It would also be interesting to explore any differences between universities with high proportions of low SES and with universities of low proportions of low SES.

Future research should also investigate the behavioural impacts of sleep hygiene on students from different social class backgrounds. Although we did not directly measure the feasibility of sleep hygiene, it is likely that perceived feasibility reflects students' actual attitudes towards implementing sleep hygiene techniques. However, simply knowing about sleep hygiene practices may not be sufficient to help improve sleep, especially for individuals from lower social class backgrounds. Indeed, our research has demonstrated that lower‐class students find sleep hygiene more difficult to implement. Therefore, future research needs to consider the impact of social class when constructing sleep interventions.

### Conclusions and implications

The results of the present research are consistent with the hypothesis that people of lower social class backgrounds have poorer mental and physical health partly because they have poorer quality sleep. Ultimately, the present studies have demonstrated a consistent and robust mediation effect of sleep on the relationship between social class and health. This mediation effect remained significant even when controlling for numerous possible confounding variables. In particular, the present research highlights that sleep quality is the most robust sleep measure and thus, a global approach to improving sleep is likely to be the most beneficial when attempting to reduce the impact that social class has on health outcomes.

The results for our first hypothesis imply that sleep needs to be a greater focus when considering the relationship between social class and health. There is robust evidence that sleep interventions can improve sleep quality (Lin et al., 2018) as well as mental health (Gee et al., 2018). Given that people from lower social class backgrounds tend to have more depressive symptoms than people from higher social class backgrounds (Demakakos et al., 2008; Rubin et al., 2016), and these may be mediated by poor sleep, non‐pharmacological sleep interventions could be useful to help improve their sleep and subsequently their mental health.

Gee et al. (2018) also highlighted that non‐pharmacological sleep interventions can be cost‐effective. As the results for our second hypothesis have shown that attempting to improve the sleep of students from lower social class backgrounds is easier if the cost impact is reduced. Thus, it is important to consider the impact that social class has when considering ways to improve sleep.

## AUTHOR CONTRIBUTIONS

**Romany McGuffog:** Conceptualization; data curation; formal analysis; investigation; methodology; project administration; validation; visualization; writing – original draft; writing – review and editing. **Mark Rubin:** Conceptualization; methodology; resources; supervision; writing – review and editing. **Mark Boyes:** Project administration; resources; writing – review and editing. **Marie L. Caltabiano:** Project administration; resources; writing – review and editing. **James Collison:** Project administration; resources; writing – review and editing. **Geoff P. Lovell:** Project administration; resources; writing – review and editing. **Orla Muldoon:** Project administration; resources; writing – review and editing. **Stefania Paolini:** Project administration; resources; writing – review and editing.

## CONFLICT OF INTEREST STATEMENT

All authors declare no conflict of interest.

### OPEN RESEARCH BADGES

This article has earned Open Data and Open Materials badges. Data and materials are available at https://osf.io/mj43c/?view_only=f8f1cf89bd244974aee577ee7d6be691.

## Supporting information


Data S1

## Data Availability

De‐identified data files are available as a part of our Supplementary Materials (https://osf.io/mj43c/?view_only=f8f1cf89bd244974aee577ee7d6be691).

## Footnotes

^1^
Please note that there were no substantive differences in the pattern of results when we used the DASS subscales (i.e. depression, anxiety and stress) rather than the global DASS score in our analyses.

^2^
In Study 1, we attempted to obtain a better understanding of the causal directions of our effects by using a longitudinal study. However, we had a very small Wave 2 sample (*N* = 57) and, thus, low power (0.30). Consequently, we were not able to appropriately investigate longitudinal effects.
