# Supplementary material for: Sleep as a mediator of the relationship between social class and health in higher education students
Source: Br J Psychol. 2023 Mar 9;114(3):710–30. doi: 10.1111/bjop.12645 (PMC10952763; doi:10.1111/bjop.12645)
Supplement: Supplementary file 1 — Data S1 [file BJOP-114-710-s001.docx]

Supplementary Materials for

**Sleep as a Mediator of the Relation between Social Class and Health**

Table of Contents

[Further Information on Measures 4](#_Toc126357413)

[Sleep Disturbance Questionnaire 4](#_Toc126357414)

[Additional Measures 7](#_Toc126357415)

[Additional Hypotheses 11](#_Toc126357416)

[Additional Results 12](#_Toc126357417)

[Preliminary Analyses 12](#_Toc126357418)

[Detailed Mediation Tests for Hypothesis 1 22](#_Toc126357419)

[Detailed Comparison of Sleep Measures as Mediators 32](#_Toc126357431)

[Detailed Correlations for Hypothesis 2 34](#_Toc126357436)

[Sleep Hygiene Serial Mediation 34](#_Toc126357439)

# **Further Information on Measures**

## Sleep Disturbance Questionnaire

This section outlines the development of the Sleep Disturbance Questionnaire. In a pilot study, 49 Australian university students answered five questions in an online study that probed the causes of any disturbances that they had when falling asleep, during sleep, and waking up. A qualitative analysis revealed eight categories of sleep disturbances, including stress/anxiety/worry, climate, stimuli, activities, poor sleep routine, pain/discomfort, bodily needs and dreams/nightmares. Based on the results of this pilot study, we developed 22 items to assess the extent to which each of these eight issues make it difficult to sleep during each of the three different stages of sleep (in the final scale, dreams/nightmares were only asked about in the during sleep and waking up stages, and activities were only asked about during the falling asleep and waking up stages).

To evaluate the psychometric properties of the Sleep Disturbance Questionnaire, we conducted correlation analyses between sleep measures across all three studies (Tables S1-S3). The results demonstrated that two sleep measures – sleep quality and presleep worries – had the strongest correlations with sleep disturbances across all studies. This result is logical because sleep quality contains items about sleep disturbances, and presleep worries are a specific type of sleep disturbance. Importantly, the correlations highlight that the Sleep Disturbance Questionnaire measures similar constructs to the sleep quality and presleep worries measures, but are not too strongly related to make the Sleep Disturbance Questionnaire redundant. On the other hand, the correlations between sleep disturbances with sleep duration, daytime sleepiness, and sleep variability were much weaker. The size of these correlations were expected because sleep disturbances is a different facet of sleep compared to sleep duration, daytime sleepiness, and sleep variability.

Table S1
*Study 1 Sleep Measures: Correlation Coefficients*

|  | Sleep quality | Daytime sleepiness | Sleep duration | Sleep disturbances | Presleep worries | Sleep schedule variability |
| --- | --- | --- | --- | --- | --- | --- |
| Sleep quality | - | - | - | - | - | - |
| Daytime sleepiness | .37^**^ | - | - | - | - | - |
| Sleep duration | - | .12^**^ | - | - | - | - |
| Sleep disturbances | .67^**^ | .32^**^ | .32^**^ | - | - | - |
| Presleep worries | .56^**^ | .31^**^ | .25^**^ | .70^**^ | - | - |
| Sleep schedule variability | .25^**^ | .18^**^ | .22^**^ | .25^**^ | .18^**^ | - |

*Note*. The response scales for daytimes sleepiness, sleep disturbances, and presleep worries ranged from 1 to 7. The response scales for sleep quality and sleep duration ranged from 0 to 3. The response scale for sleep schedule variability ranged from 1 to 6.

* = *p* <.05. ** = *p* < .01.

Table S2
*Study 2 Sleep Measures: Correlation Coefficients*

|  | Sleep quality | Daytime sleepiness | Sleep duration | Sleep disturbances | Presleep worries | Sleep schedule variability |
| --- | --- | --- | --- | --- | --- | --- |
| Sleep quality | - | - | - | - | - | - |
| Daytime sleepiness | .37^*^ | - | - | - | - | - |
| Sleep duration | - | .13^*^ | - | - | - | - |
| Sleep disturbances | .61^**^ | .32^**^ | .27^**^ | - | - | - |
| Presleep worries | .58^**^ | .31^**^ | .26^**^ | .69^**^ | - | - |
| Sleep schedule variability | .37^**^ | .24^**^ | .16^**^ | .30^**^ | .18^**^ | - |

*Note*. The response scales for daytimes sleepiness, sleep disturbances, and presleep worries ranged from 1 to 7. The response scales for sleep quality and sleep duration ranged from 0 to 3. The response scale for sleep schedule variability ranged from 1 to 6.

* = *p* <.05. ** = *p* < .01.

Table S3
*Study 3 Sleep Measures: Correlation Coefficients*

|  | Sleep quality | Daytime sleepiness | Sleep duration | Sleep disturbances | Presleep worries | Sleep schedule variability |
| --- | --- | --- | --- | --- | --- | --- |
| Sleep quality | - | - | - | - | - | - |
| Daytime sleepiness | .32^**^ | - | - | - | - | - |
| Sleep duration | - | .10^*^ | - | - | - | - |
| Sleep disturbances | .63^**^ | .24^**^ | .24^**^ | - | - | - |
| Presleep worries | .57^**^ | .25^**^ | .23^**^ | .67^**^ | - | - |
| Sleep schedule variability | .24^**^ | .13^**^ | .15^**^ | .25^**^ | .32^**^ | - |

*Note*. The response scales for daytimes sleepiness, sleep disturbances, and presleep worries ranged from 1 to 7. The response scale for sleep quality ranged from 0 to 3. The response scale for sleep schedule variability ranged from 1 to 6. The response scale for sleep duration ranged from 0 to 24.

* = *p* <.05. ** = *p* < .01.

## Additional Measures

In each study, there were additional measures that were not included in the presented research. In this section, we detail these additional measures. Unless otherwise specified, the response scales for each measure *strongly disagree* (1) to *strongly agree* (7).

***Measures Included Across All Studies***

We measured social contact using a variation of the social contact scale developed by Rubin et al. (2016). We included six items from the scale which assessed the number of friends that students communicated with during the past week via (i) face-to-face meetings, (ii) text message, (iii) Facebook, (iv) Twitter, (v) Snapchat, and (vi) WhatsApp. Responses were made on a scale ranging from *0* to *more than 10*.

Another non-sleep mediator we considered was diet quality. Darmon and Drewnowski (2008) suggested that lower quality diets were more likely to be consumed by less affluent people, and that higher quality diets were more likely to be consumed by more affluent people. Therefore, to measure diet quality, we created four items which measured the healthiness of participants’ diet (*extremely unhealthy* to *extremely healthy*) and their regularity of meals (*extremely irregular* to *extremely regular*). We created these items as a short alternative to the long and detailed measures that are often used to measure diet (Hurley et al., 2009).

Finally, we created a single item to measure caffeine intake based on Jarvis (1993); “On average, how many caffeinated drinks do you consume (e.g., coffee, tea, energy drinks, coke)?” The 6-point response scale ranged from *less than one a week* (1) to *more than 2-3 a day* (6).

***Measures only Included in Study 1***

In Study 1, we included a two-item measure of sense of belonging based of Rubin and Wright’s (2015) scale. An example item is “Over the past four weeks, I felt a sense of belonging at university.”

***Measures only Included in Study 2***

For Study 2, to measure social support, we included the 24-item Social Provisions Scale (Cutrona & Russell, 1987). This scale includes six subscales that includes guidance, reassurance of worth, social integration, attachment, nurturance, and reliable alliance. An example item includes “I feel that I do not have close personal relationships with other people.” This scale demonstrated a good internal reliability, with a Cronbach α of .92 in Cutrona and Russell’s study. The researchers also reported that the scale demonstrated good construct validity by being positively related to a measure of interpersonal relationships and negatively related to a measure of loneliness.

In Study 2, to investigate the role of dreams as a factor of sleep, we included a modified version of Nielsen et al.’s (2003) Typical Dreams Questionnaire. This questionnaire measures the frequency of particular types of dreams by asking participants how often they recall dreaming about certain themes. The scale included 17 items, such as “childhood memories” and “falling or flying”. Responses ranged from *never* (1) to *all the time* (7).

Based on the aspects of dreams and nightmares reported in previous literature (e.g., Nielson et al., 2006; Zadra, & Donderi; 2000), Study 2 also included six items about the frequency of dreams and nightmares, how much dreams and nightmares impact on people once they have woken, and how vividly people can recall their dreams and nightmares. An example item of the frequency of dreams and nightmares is: “Over the past seven days, how often have you had nightmares/bad dreams?” The response scale ranged from *0 days* (0) to *7 days* (7). An example item of the impact of dreams and nightmares is: “Over the past seven days, on average, to what extent do your dreams impact or affect you after you have woken up?” The response scale ranged from *not at all* (1) to *a great deal* (7). Finally, an example item of the recall of dreams and nightmares is: “Think of your most recent nightmares/bad dreams, how vividly can you recall the contents of these nightmares/bad dreams?” The response scale ranged from *not at all* (1) to *a great deal* (7).

Following Zadra et al.’s (2006) work, which found that fear was the most predominant emotion experienced in nightmares/bad dreams, Study 2 also included a single item to measure the intensity of fear experienced in nightmares/bad dreams: “Thinking about your most recent nightmares/bad dreams, how intense was your fear?” Responses ranged from *very weak* (1) to *very intense* (9).

Furthermore, we measured death anxiety by including Florian and Mikulincer’s (1997) 4-item Self-Annihilation scale. An example item is: “Death frightens me because of the loss and destruction of myself.” Participants responded using a 7-point response scale ranging from *strongly disagree* (1) to *strongly agree* (7). This measure was related to a separate theoretical question which was not explored in the current article.

***Measures only Included in Study 3***

For Study 3, we included additional prospective mediators based on the literature investigating explanations for the relationship between social class and health. First, we included the 10-item General Self-Efficacy Scale by Schwarzer and Jerusalem (1995). We modified the response scale from *not at all true* (1) to *exactly true* (4) to a 7-point scale ranging from *strongly disagree* (1) to *strongly agree* (7). An example item is “it is easy for me to stick to my aims and accomplish my goals.”

We also added two items related to the safety of neighbourhoods and violence experienced in neighbourhoods from Johnson et al. (2009). An example item is “how often have you felt safe walking alone during the day?” with a 7-point response scale ranging from *never* (1) to *all the time* (7). We added three items measuring neighbourbood social capital such as “I live in a close-knit neighbourhood,” which were adapted from Sampson et al. (1997).

To measure the accessibility to resources, we created a 7-item scale which asked participants whether they had access to various resources such as “places for recreation (such as parks, walking tracks, playgrounds, etc.)” and “grocery shops (such as Coles, Woolworths, IGA, etc.).” We also created two items to measure family conflict, including “growing up, there was a lot of conflict between household members in my family home” (for childhood family conflict) and “over the past four weeks, there has been a lot of conflict between the people that I live with” (for current family conflict).

We used four items from Rubin and Kelly’s (2015) parenting styles measure. An example item included “my parents praised me when I was good.” A higher score on these items indicates more authoritative parenting, including more parental warmth, responsiveness to children’s needs, and respect for children’s autonomy.

We also included a shortened version of the Brief COPE. Carver (1997) created the Brief COPE with only 28 items, consisting of 14 types of coping strategies (thus, two items per coping strategy). The strategies include self-distraction, active coping, denial, substance use, use of emotional support, use of instrumental support, behavioural disengagement, venting, positive reframing, planning, humour, acceptance, religion, and self-blame. However, we wanted an even shorter version to keep the length of my survey as short as possible so as to retain participants. Therefore, we used the highest loading item from each of the 14 factors identified in the factor analysis by Monzani et al. (2015), which provided support for the factor structure suggested by Carver. Thus, the item count for the measure was reduced to 14 items. The scale asked participants to consider how they have been coping with recent problems or obstacles, and indicated the extent to which they have used each coping strategy (e.g., “I’ve been taking action to try and make the situation better”).

As a measure of decision-making, we included an adapted version of the Domain-Specific Risk-Taking (Adult) Scale (Blais & Weber, 2006), which asked participants to indicate the likelihood of engaging in certain activities. We selected the items that referred to health and safety risks because these were the most relevant items to the present research. Additionally, we avoided items that involved financial elements, because these items were too proximal to the social class items. We used a total of nine items to measure health and safety risk-taking, including “ignoring some persistent physical pain by not going to the doctor” and “driving a car without wearing a seatbelt.” Participants responded on a 5-point scale ranging from *extremely unlikely* (1) to *extremely likely* (5).

Finally, we also included two additional diet items; skipping meals (*never* to *all the time*), and snacking between meals (*never* to *all the time*).

***Measures only Included in Studies 2 and 3***

In Studies 2 and 3, we included a shortened version of Terrill et al.’s (2015) Background Stress Inventory. This measure contains five items which measure the distress associated with a variety of issues. An example item is: “How distressed have you felt by work-related issues (e.g., commuting, deadlines, promotions, feeling appreciated, getting work done)?” Participants responded using a 7-point response scale ranging from *never* (1) to *all the time* (7).We also included three additional items related to diet, including their regularity of meals (extremely irregular to extremely regular), skipping meals (never to all the time), and snacking between meals (never to all the time).

# **Additional Hypotheses**

We also aimed to explore whether social class identification moderated the relations between social class, sleep, and health. In all three studies, we also included a measure of how much the participants identify with their social class, using a measure of in-group identification by Leach et al. (2008) which was adapted to social class by Rubin and Stuart (2017). We included three subscales from this measure which each contain two items. The subscales look at perceived self-class similarity, importance of identity, and salience of identity. One item in each subscale was reversed scored. An example item is “I am quite similar to the other people in my social class.” Responses were made on a 7-point scale ranging from strongly disagree (1) to strongly agree (7). We used a split-half reliability test to check the internal reliability of the subscales, following Eisinga et al. (2013). We investigated the Spearman-Brown Coefficient for perceived self-class similarity (range = .62 to .79), importance of identity (range = .47 to .73), and salience of identity (range = .72 to .79). We conducted moderation analyses to investigate whether (a) higher levels of perceived self-to-class similarity decrease the positive association between social class and (i) sleep and (ii) mental and physical health, whereas (b) higher levels of perceived importance and salience of social class increase the size of these positive associations. In general, these moderation tests did not yield any significant results. These null results may have occurred either due to a Type I error in the original study or a Type II error in this study, or an incorrect theoretical inference.

# **Additional Results**

## Preliminary Analyses

***Normality***

Normality was checked before conducting any analyses. Across all three studies, all variables were normally distributed with only minor exceptions. The skewness and kurtosis for age across each study was greater than ± 2.0. To address this non-normality, we removed univariate outliers (> ±3 SD) for age, which reduced the skewness to below ± 2.0 in Studies 1 and 2. However, the skewness in Study 3 remained high (2.23), as did the kurtosis across all studies (ranging from 2.71 to 4.86). Despite these issues, age was a covariate variable and not a key variable, so we considered this improvement in normality to be sufficient. However, it is important to note that any results involving these variables need to be interpreted with caution. In addition, in Study 3, sleep duration had skewness and kurtosis greater than ± 2.0. However, when univariate outliers for sleep duration were removed, the skewness and kurtosis were reduced to below ± 2.0. Throughout all three studies, age was used with outliers removed, and sleep duration was used in Study 3 with outliers removed.

***Social Class Item Frequencies***

The frequencies of each of the social class items are presented in the tables below.

*Table S4*

*Frequencies of Parental Educational Level Items Across All Studies*

|  | Study 1 | | Study 2 | | Study 3 | |
| --- | --- | --- | --- | --- | --- | --- |
|  | Mother’s education | Father’s education | Mother’s education | Father’s education | Mother’s education | Father’s education |
| Primary school (Kindergarten to Year 6) | 16 (2.55%) | 15 (2.39%) | 0 (0.00%) | 3 (0.80%) | 4 (0.90%) | 4 (0.90%) |
| Secondary or high school (Years 7 to 9) | 31 (4.94%) | 53 (8.44%) | 25 (6.65%) | 31 (8.24%) | 24 (5.38%) | 38 (8.52%) |
| School Certificate / Intermediate Year / Year 10 / 4th Form | 131 (20.86%) | 131 (20.86%) | 82 (21.81%) | 88 (23.40%) | 79 (17.71%) | 105 (23.54%) |
| HSC / Leaving / Year 12 / 6th Form | 117 (18.63%) | 113 (17.99%) | 65 (17.29%) | 48 (12.77%) | 79 (17.71%) | 72 (16.14%) |
| Technical and Further Education (TAFE) Certificate or Diploma | 133 (21.18%) | 147 (23.41%) | 83 (22.07%) | 94 (25.00%) | 116 (26.01%) | 115 (25.78%) |
| University or College of Advanced Education - undergraduate degree (Bachelor degree) | 149 (23.73%) | 110 (17.52%) | 98 (26.06%) | 74 (19.68%) | 102 (22.87%) | 71 (15.92%) |
| University or College of Advanced Education - postgraduate degree (Masters or PhD) | 51 (8.12%) | 59 (9.39%) | 23 (6.12%) | 38 (10.11%) | 42 (9.42%) | 41 (9.19%) |

*Table S5*

*Frequencies of Parental Occupation Prestige and Status Items Across All Studies*

|  | Study 1 | | Study 2 | | Study 3 | |
| --- | --- | --- | --- | --- | --- | --- |
|  | Status and prestige of mother’s occupation | Status and prestige of father’s education | Status and prestige of mother’s occupation | Status and prestige of father’s education | Status and prestige of mother’s occupation | Status and prestige of father’s education |
| Extremely low status and prestige | 14 (2.23%) | 15 (2.39%) | 13 (3.46%) | 9 (2.39%) | 6 (1.35%) | 13 (2.91%) |
| Very low status and prestige | 15 (2.39%) | 15 (2.39%) | 17 (4.52%) | 12 (3.19%) | 16 (3.59%) | 16 (3.59%) |
| Low status and prestige | 58 (9.24%) | 54 (8.60%) | 30 (7.98%) | 27 (7.18%) | 44 (9.87%) | 26 (5.83%) |
| Moderately status and prestige | 86 (13.69%) | 50 (7.96%) | 36 (9.57%) | 33 (8.78%) | 51 (11.43%) | 42 (9.42%) |
| Slightly below average status and prestige | 33 (5.25%) | 23 (3.66%) | 22 (5.85%) | 20 (5.32%) | 32 (7.17%) | 34 (7.62%) |
| Average status and prestige | 156 (24.84%) | 149 (23.73%) | 98 (26.06%) | 76 (20.21%) | 117 (26.23%) | 102 (22.87%) |
| Slightly above average status and prestige | 94 (14.97%) | 92 (14.65%) | 54 (14.36%) | 51 (13.56%) | 63 (14.13%) | 55 (12.33%) |
| Moderately above average status and prestige | 98 (15.61%) | 118 (18.79%) | 60 (15.96%) | 70 (18.62%) | 69 (15.47%) | 64 (14.35%) |
| High status and prestige | 48 (7.64%) | 79 (12.58%) | 37 (9.84%) | 48 (12.77%) | 31 (6.95%) | 56 (12.56%) |
| Very high status and prestige | 21 (3.34%) | 27 (4.30%) | 7 (1.86%) | 22 (5.85%) | 13 (2.91%) | 28 (6.28%) |
| Extremely high status and prestige | 5 (0.80%) | 6 (0.96%) | 2 (0.53%) | 8 (2.13%) | 4 (0.90%) | 10 (2.24%) |

*Table S6*

*Frequencies of Childhood Wealth Items Across All Studies*

|  | Study 1 | | | Study 2 | | | Study 3 | | |
| --- | --- | --- | --- | --- | --- | --- | --- | --- | --- |
|  | My family usually had enough money to buy things when I was growing up. | I felt relatively wealthy compared to the other kids in my high school. | I grew up in a relatively wealthy neighbourhood. | My family usually had enough money to buy things when I was growing up. | I felt relatively wealthy compared to the other kids in my high school. | I grew up in a relatively wealthy neighbourhood. | My family usually had enough money to buy things when I was growing up. | I felt relatively wealthy compared to the other kids in my high school. | I grew up in a relatively wealthy neighbourhood. |
| Strongly disagree | 25 (3.98%) | 67 (10.67%) | 56 (8.92%) | 13 (3.46%) | 34 (9.04%) | 29 (7.71%) | 13 (2.91%) | 42 (9.42%) | 33 (7.40%) |
| Disagree | 60 (9.55%) | 125 (19.90%) | 120 (19.11%) | 34 (9.04%) | 72 (19.15%) | 51 (13.56%) | 26 (5.83%) | 79 (17.71%) | 69 (15.47%) |
| Partially disagree | 61 (9.71%) | 99 (15.76%) | 98 (15.61%) | 34 (9.04%) | 63 (16.76%) | 56 (14.89%) | 47 (10.54%) | 67 (15.02%) | 57 (12.78%) |
| Neutral | 54 (8.60%) | 164 (26.11%) | 128 (20.38%) | 27 (7.18%) | 99 (26.33%) | 77 (20.48%) | 33 (7.40%) | 93 (20.85%) | 95 (21.30%) |
| Partially agree | 126 (20.06%) | 98 (15.61%) | 121 (19.27%) | 76 (20.21%) | 53 (14.10%) | 78 (20.74%) | 95 (21.30%) | 85 (19.06%) | 81 (18.16%) |
| Agree | 216 (34.39%) | 59 (9.39%) | 89 (14.17%) | 127 (33.78%) | 43 (11.44%) | 67 (17.82%) | 161 (36.10%) | 63 (14.13%) | 86 (19.28%) |
| Strongly agree | 86 (13.69%) | 16 (2.55%) | 16 (2.55%) | 65 (17.29%) | 12 (3.19%) | 18 (4.79%) | 71 (15.92%) | 17 (3.81%) | 25 (5.61%) |

*Table S7*

*Frequencies of Subjective Social Class Items Across All Studies*

|  | Study 1 | | | Study 2 | | | Study 3 | | |
| --- | --- | --- | --- | --- | --- | --- | --- | --- | --- |
|  | Individual’s social class | Mother’s social class | Father’s social class | Individual’s social class | Mother’s social class | Father’s social class | Individual’s social class | Mother’s social class | Father’s social class |
| Working-class | 79 (12.58%) | 99 (15.76%) | 123 (19.59%) | 51 (13.56%) | 65 (17.29%) | 77 (20.48%) | 59 (13.23%) | 93 (20.85%) | 114 (25.56%) |
| Lower middle-class | 103 (16.40%) | 118 (18.79%) | 87 (13.85%) | 62 (16.49%) | 68 (18.09%) | 60 (15.96%) | 77 (17.26%) | 84 (18.83%) | 80 (17.94%) |
| Middle-class | 318 (50.64%) | 286 (45.54%) | 280 (44.59%) | 189 (50.27%) | 175 (46.54%) | 156 (41.49%) | 195 (43.72%) | 187 (41.93%) | 171 (38.34%) |
| Upper middle-class | 124 (19.75%) | 120 (19.11%) | 129 (20.54%) | 73 (19.41%) | 63 (16.76%) | 75 (19.95%) | 113 (25.34%) | 76 (17.04%) | 74 (16.59%) |
| Upper class | 4 (0.64%) | 5 (0.80%) | 9 (1.43%) | 1 (0.27%) | 5 (1.33%) | 8 (2.13%) | 2 (0.45%) | 6 (1.35%) | 7 (1.57%) |

*Table S8*

*Frequencies of MacArthur Subjective Social Status Scale Across All Studies*

|  | Study 1 | Study 2 | Study 3 |
| --- | --- | --- | --- |
| Top Level | 4 (0.64%) | 1 (0.27%) | 1 (0.22%) |
| Level 9 | 9 (1.43%) | 8 (2.13%) | 6 (1.35%) |
| Level 8 | 38 (6.05%) | 29 (7.71%) | 43 (9.64%) |
| Level 7 | 105 (16.72%) | 73 (19.41%) | 105 (23.54%) |
| Level 6 | 124 (19.75%) | 74 (19.68%) | 93 (20.85%) |
| Middle Level | 212 (33.76%) | 99 (26.33%) | 97 (21.75%) |
| Level 4 | 65 (10.35%) | 42 (11.17%) | 49 (10.99%) |
| Level 3 | 43 (6.85%) | 28 (7.45%) | 30 (6.73%) |
| Level 2 | 14 (2.23%) | 12 (3.19%) | 14 (3.14%) |
| Level 1 | 9 (1.43%) | 6 (1.60%) | 4 (0.90%) |
| Bottom Level | 5 (0.80%) | 4 (1.06%) | 4 (0.90%) |

***Social Class Items Intercorrelations***

Across all three studies, we combined the 11 social class items into a single global scale. Along with a factor analysis (which was presented in the main paper), we also explored the intercorrelations between the social class items in Study 1 (Table S9), Study 2 (Table S10), and Study 3 (Table S11).

*Table S9*

*Intercorrelations Between Social Class Items (Study 1)*

|  | Mother’s education | Father’s education | Mother’s job prestige | Father’s job prestige | Childhood wealth 1 | Childhood wealth 2 | Childhood wealth 3 | Individual’s social class | Mother’s social class | Father’s social class | MacArthur scale |
| --- | --- | --- | --- | --- | --- | --- | --- | --- | --- | --- | --- |
| Mother’s education | - | .36^**^ | .40^**^ | .08^*^ | .16^*^ | .12^**^ | .27^**^ | .16^**^ | .31^**^ | .19^**^ | .17^**^ |
| Father’s education | - | - | .13^**^ | .38^**^ | .17^**^ | .18^**^ | .24^**^ | .20^**^ | .26^**^ | .38^**^ | .15^**^ |
| Mother’s job prestige | - | - | - | .36^**^ | .29^**^ | .28^**^ | .24^**^ | .26^**^ | .45^**^ | .24^**^ | .28^**^ |
| Father’s job prestige | - | - | - | - | .32^**^ | .32^**^ | .28^**^ | .29^**^ | .29^**^ | .47^**^ | .29^**^ |
| Childhood wealth 1 | - | - | - | - | - | .58^**^ | .49^**^ | .35^**^ | .35^**^ | .38^**^ | .28^**^ |
| Childhood wealth 2 | - | - | - | - | - | - | .51^**^ | .36^**^ | .34^**^ | .37^**^ | .30^**^ |
| Childhood wealth 3 | - | - | - | - | - | - | - | .37^**^ | .38^**^ | .42^**^ | .32^**^ |
| Individual’s social class | - | - | - | - | - | - | - | - | .59^**^ | .54^**^ | .53^**^ |
| Mother’s social class | - | - | - | - | - | - | - | - | - | .59^**^ | .37^**^ |
| Father’s social class | - | - | - | - | - | - | - | - | - | - | .31^**^ |
| MacArthur scale | - | - | - | - | - | - | - | - | - | - | - |

**p* < .05. ***p* < .01.

*Table S10*

*Intercorrelations Between Social Class Items (Study 2)*

|  | Mother’s education | Father’s education | Mother’s job prestige | Father’s job prestige | Childhood wealth 1 | Childhood wealth 2 | Childhood wealth 3 | Individual’s social class | Mother’s social class | Father’s social class | MacArthur scale |
| --- | --- | --- | --- | --- | --- | --- | --- | --- | --- | --- | --- |
| Mother’s education | - | .41^**^ | .47^**^ | .20^*^ | .15^**^ | .12^*^ | .21^**^ | .18^**^ | .29^**^ | .16^**^ | .22^**^ |
| Father’s education | - | - | .24^**^ | .41^**^ | .15^**^ | .13^*^ | .22^**^ | .19^**^ | .16^**^ | .35^**^ | .21^**^ |
| Mother’s job prestige | - | - | - | .39^**^ | .33^**^ | .35^**^ | .30^**^ | .37^**^ | .44^**^ | .30^**^ | .36^**^ |
| Father’s job prestige | - | - | - | - | .37^**^ | .41^**^ | .36^**^ | .40^**^ | .28^**^ | .52^**^ | .40^**^ |
| Childhood wealth 1 | - | - | - | - | - | .62^**^ | .54^**^ | .37^**^ | .29^**^ | .28^**^ | .38^**^ |
| Childhood wealth 2 | - | - | - | - | - | - | .64^**^ | .45^**^ | .36^**^ | .38^**^ | .42^**^ |
| Childhood wealth 3 | - | - | - | - | - | - | - | .38^**^ | .32^**^ | .36^**^ | .33^**^ |
| Individual’s social class | - | - | - | - | - | - | - | - | .66^**^ | .59^**^ | .62^**^ |
| Mother’s social class | - | - | - | - | - | - | - | - | - | .59^**^ | .38^**^ |
| Father’s social class | - | - | - | - | - | - | - | - | - | - | .44^**^ |
| MacArthur scale | - | - | - | - | - | - | - | - | - | - | - |

**p* < .05. ***p* < .01.

*Table S11*

*Intercorrelations Between Social Class Items (Study 3)*

|  | Mother’s education | Father’s education | Mother’s job prestige | Father’s job prestige | Childhood wealth 1 | Childhood wealth 2 | Childhood wealth 3 | Individual’s social class | Mother’s social class | Father’s social class | MacArthur scale |
| --- | --- | --- | --- | --- | --- | --- | --- | --- | --- | --- | --- |
| Mother’s education | - | .48^**^ | .41^**^ | .10^*^ | .14^**^ | .16^**^ | .23^**^ | .23^**^ | .29^**^ | .07 | .18^**^ |
| Father’s education | - | - | .14^**^ | .36^**^ | .20^**^ | .25^**^ | .33^**^ | .27^**^ | .22^**^ | .31^**^ | .19^**^ |
| Mother’s job prestige | - | - | - | .35^**^ | .33^**^ | .29^**^ | .28^**^ | .37^**^ | .40^**^ | .21^**^ | .32^**^ |
| Father’s job prestige | - | - | - | - | .35^**^ | .41^**^ | .43^**^ | .47^**^ | .30^**^ | .48^**^ | .37^**^ |
| Childhood wealth 1 | - | - | - | - | - | .65^**^ | .55^**^ | .40^**^ | .37^**^ | .35^**^ | .30^**^ |
| Childhood wealth 2 | - | - | - | - | - | - | .63^**^ | .50^**^ | .42^**^ | .37^**^ | .37^**^ |
| Childhood wealth 3 | - | - | - | - | - | - | - | .39^**^ | .40^**^ | .34^**^ | .28^**^ |
| Individual’s social class | - | - | - | - | - | - | - | - | .57^**^ | .56^**^ | .55^**^ |
| Mother’s social class | - | - | - | - | - | - | - | - | - | .61^**^ | .34^**^ |
| Father’s social class | - | - | - | - | - | - | - | - | - | - | .30^**^ |
| MacArthur scale | - | - | - | - | - | - | - | - | - | - | - |

**p* < .05. ***p* < .01.

## Detailed Mediation Tests for Hypothesis 1

In addition to the mediation tests presented in the main manuscript of sleep quality across all three studies, we present the other mediation tests in this section.

Table S12
*The Mediating Effect of Sleep Disturbances on the Relationship Between Social Class and Each Outcome Variable (Study 1)*

| Outcome variables | Effect type | *b* (*SE*) | 95% CIs | *t* | *p* | CSIES (Reverse) |
| --- | --- | --- | --- | --- | --- | --- |
| Physical health | Total | -.19 (.07) | -0.33, -0.05 | -2.61 | .009 |  |
| symptoms | Direct | -.11 (.06) | -0.22, 0.01 | -1.77 | .077 |  |
|  | Indirect | -.08 (.04) | -0.16, -0.01 | - | - | -.05 (-.06) |
|  |  |  |  |  |  |  |
| General physical | Total | -.44 (.07) | -0.57, -0.30 | -6.38 | <.001 |  |
| health | Direct | -.38 (.06) | -0.50, -0.26 | -6.06 | <.001 |  |
|  | Indirect | -.06 (.03) | -0.12, -0.003 | - | - | -.03 (-.10) |
|  |  |  |  |  |  |  |
| General distress | Total | -.16 (.04) | -0.23, -0.08 | -4.25 | <.001 |  |
|  | Direct | -.11 (.03) | -0.17, -0.05 | -3.75 | <.001 |  |
|  | Indirect | -.04 (.02) | -0.08, -0.002 | - | - | -.05 (-.10) |
|  |  |  |  |  |  |  |
| Self-esteem | Total | .49 (.10) | 0.28, 0.69 | 4.64 | <.001 |  |
|  | Direct | .42 (.10) | 0.23, 0.62 | 4.21 | <.001 |  |
|  | Indirect | .06 (.03) | 0.01, 0.13 | - | - | .02 (-.06) |

*Note.* Presleep worries is the mediator variable for all models. All Models have Dfs of 2, 625. See the Table 1 note in the main paper for further details.

Table S13
*The Mediating Effect of Sleep Duration on the Relationship Between Social Class and Each Outcome Variable (Study 1)*

| Outcome variables | Effect type | *b* (*SE*) | 95% CIs | *t* | *P* | CSIES (Reverse) |
| --- | --- | --- | --- | --- | --- | --- |
| Physical health | Total | -.19 (.07) | -0.33, -0.05 | -2.61 | .009 |  |
| symptoms | Direct | -.14 (.07) | -0.28, 0.04 | -1.91 | .056 |  |
|  | Indirect | -.05 (.02) | -0.09, -0.02 | - | - | -.03 (-.02) |
|  |  |  |  |  |  |  |
| General physical | Total | -.44 (.07) | -0.57, -0.30 | -6.38 | <.001 |  |
| health | Direct | -.37 (.07) | -0.50, -0.23 | -5.39 | <.001 |  |
|  | Indirect | -.07 (.02) | -0.12, -0.04 | - | - | -.04 (-.06) |
|  |  |  |  |  |  |  |
| General distress | Total | -.16 (.04) | -0.23, -0.08 | -4.25 | <.001 |  |
|  | Direct | -.12 (.04) | -0.19, -0.05 | -3.27 | .001 |  |
|  | Indirect | -.04 (.01) | -0.06, -0.02 | - | - | -.04 (-.04) |
|  |  |  |  |  |  |  |
| Self-esteem | Total | .49 (.10) | 0.28, 0.69 | 4.64 | <.001 |  |
|  | Direct | .42 (.11) | 0.21, 0.63 | 3.99 | <.001 |  |
|  | Indirect | .07 (.02) | 0.03, 0.12 | - | - | .02 (-.02) |

*Note.* Sleep duration is the mediator variable for all models. All Models have Dfs of 2, 625. See the Table 1 note in the main paper for further details.

Table S14
*The Mediating Effect of Presleep Worries on the Relationship Between Social Class and Each Outcome Variable (Study 1)*

| Outcome variables | Effect type | *b* (*SE*) | 95% CIs | *t* | *P* | CSIES (Reverse) |
| --- | --- | --- | --- | --- | --- | --- |
| Physical health | Total | -.19 (.07) | -0.33, -0.05 | -2.61 | .009 |  |
| symptoms | Direct | -.08 (.06) | -0.20, 0.04 | -1.25 | .212 |  |
|  | Indirect | -.11 (.04) | -0.19, -0.04 | - | - | -.06 (-.05) |
|  |  |  |  |  |  |  |
| General physical | Total | -.44 (.07) | -0.57, -0.30 | -6.38 | <.001 |  |
| health | Direct | -.36 (.06) | -0.48, -0.23 | -5.60 | <.001 |  |
|  | Indirect | -.08 (.03) | -0.14, -0.03 | - | - | -.05 (-.10) |
|  |  |  |  |  |  |  |
| General distress | Total | -.16 (.04) | -0.23, -0.08 | -4.25 | <.001 |  |
|  | Direct | -.09 (.03) | -0.15, -0.03 | -3.03 | .003 |  |
|  | Indirect | -.07 (.02) | -0.11, -0.02 | - | - | -.07 (-.10) |
|  |  |  |  |  |  |  |
| Self-esteem | Total | .49 (.10) | 0.28, 0.69 | 4.64 | <.001 |  |
|  | Direct | .38 (.10) | 0.18, 0.57 | 3.81 | <.001 |  |
|  | Indirect | .11 (.04) | 0.03, 0.20 | - | - | .04 (-.06) |

*Note.* Presleep worries is the mediator variable for all models. All Models have Dfs of 2, 625. See the Table 1 note in the main paper for further details.

Table S15
*The Mediating Effect of Sleep Schedule Variability on the Relationship Between Social Class and Each Outcome Variable (Study 1)*

| Outcome variables | Effect type | *b* (*SE*) | 95% CIs | *t* | *P* | CSIES (Reverse) |
| --- | --- | --- | --- | --- | --- | --- |
| Physical health | Total | -.19 (.07) | -0.33, -0.05 | -2.61 | .009 |  |
| symptoms | Direct | -.17 (.07) | -0.31, -0.03 | -2.36 | .019 |  |
|  | Indirect | -.02 (.01) | -0.05, -0.002 | - | - | -.01 (-.01) |
|  |  |  |  |  |  |  |
| General physical | Total | -.44 (.07) | -0.57, -0.30 | -6.38 | <.001 |  |
| health | Direct | -.40 (.07) | -0.54, -0.27 | -5.95 | <.001 |  |
|  | Indirect | -.04 (.02) | -0.08, -0.01 | - | - | -.02 (-.05) |
|  |  |  |  |  |  |  |
| General distress | Total | -.16 (.04) | -0.23, -0.08 | -4.25 | <.001 |  |
|  | Direct | -.14 (.04) | -0.21, -0.07 | -3.88 | <.001 |  |
|  | Indirect | -.01 (.01) | -0.03, -0.002 | - | - | -.02 (-.03) |
|  |  |  |  |  |  |  |
| Self-esteem | Total | .49 (.10) | 0.28, 0.69 | 4.64 | <.001 |  |
|  | Direct | .44 (.10) | 0.24, 0.65 | 4.25 | <.001 |  |
|  | Indirect | .04 (.02) | 0.01, 0.10 | - | - | .02 (-.03) |

*Note.* Sleep schedule variability is the mediator variable for all models. All Models have Dfs of 2, 625. See the Table 1 note in the main paper for further details.

Table S16
*The Mediating Effect of Sleep Duration on the Relationship Between Social Class and Each Outcome Variable (Study 2)*

| Outcome variables | Effect type | *b* (*SE*) | 95% CIs | *t* | *P* | CSIES (Reverse) |
| --- | --- | --- | --- | --- | --- | --- |
| Physical health | Total | -.23 (.09) | -0.41, -0.06 | -2.58 | .010 |  |
| symptoms | Direct | -.19 (.09) | -0.37, -0.01 | -2.12 | .034 |  |
|  | Indirect | -.04 (.02) | -0.10, -0.01 | - | - | -.02 (-.02) |
|  |  |  |  |  |  |  |
| General physical | Total | -.40 (.09) | -0.59, -0.22 | -4.26 | <.001 |  |
| health | Direct | -.38 (.10) | -0.56, -0.19 | -3.96 | <.001 |  |
|  | Indirect | -.03 (.02) | -0.07, -0.003 | - | - | -.01 (-.02) |
|  |  |  |  |  |  |  |
| General distress | Total | -.15 (.05) | -0.24, -0.06 | -3.37 | <.001 |  |
|  | Direct | -.12 (.04) | -0.21, -0.03 | -2.76 | .006 |  |
|  | Indirect | -.03 (.01) | -0.06, -0.01 | - | - | -.03 (-.05) |

*Note.* Sleep duration is the mediator variable for all models. All Models have Dfs of 2, 373. See the Table 1 note in the main paper for further details.

Table S17
*The Mediating Effect of Presleep Worries on the Relationship Between Social Class and Each Outcome Variable (Study 2)*

| Outcome variables | Effect type | *b* (*SE*) | 95% CIs | *t* | *P* | CSIES (Reverse) |
| --- | --- | --- | --- | --- | --- | --- |
| Physical health | Total | -.23 (.09) | -0.41, -0.06 | -2.58 | .010 |  |
| symptoms | Direct | -.14 (.08) | -0.29, 0.02 | -1.74 | .083 |  |
|  | Indirect | -.10 (.05) | -0.20, -0.004 | - | - | -.05 (-.07) |
|  |  |  |  |  |  |  |
| General physical | Total | -.40 (.09) | -0.59, -0.22 | -4.26 | <.001 |  |
| health | Direct | -.34 (.09) | -0.52, -0.16 | -3.76 | <.001 |  |
|  | Indirect | -.06 (.03) | -0.13, -0.002 | - | - | -.03 (-.07) |
|  |  |  |  |  |  |  |
| General distress | Total | -.15 (.05) | -0.24, -0.06 | -3.37 | <.001 |  |
|  | Direct | -.09 (.04) | -0.16, -0.16 | -2.61 | <.001 |  |
|  | Indirect | -.06 (.03) | -0.12, -0.002 | - | - | -.07 (-.11) |
|  |  |  |  |  |  |  |
| Self-esteem | Total | .44 (.14) | 0.17, 0.71 | 3.20 | .002 |  |
|  | Direct | .34 (.13) | 0.08, 0.59 | 2.60 | .010 |  |
|  | Indirect | .10 (.05) | 0.01, 0.22 | - | - | .04 (-.06) |

*Note.* Presleep worries is the mediator variable for all models. All Models have Dfs of 2, 373. See the Table 1 note in the main paper for further details.

Table S18
*The Mediating Effect of Sleep Schedule Variability on the Relationship Between Social Class and Each Outcome Variable (Study 2)*

| Outcome variables | Effect type | *b* (*SE*) | 95% CIs | *t* | *P* | CSIES (Reverse) |
| --- | --- | --- | --- | --- | --- | --- |
| Physical health | Total | -.23 (.09) | -0.41, -0.06 | -2.58 | .010 |  |
| symptoms | Direct | -.21 (.09) | -0.39, -0.03 | -2.32 | .021 |  |
|  | Indirect | -.02 (.02) | -0.07, -0.002 | - | - | -.01 (-.02) |
|  |  |  |  |  |  |  |
| General physical | Total | -.40 (.09) | -0.59, -0.22 | -4.26 | <.001 |  |
| health | Direct | -.36 (.09) | -0.54, -0.18 | -3.87 | <.001 |  |
|  | Indirect | -.04 (.02) | -0.10, -0.005 | - | - | -.02 (-.05) |
|  |  |  |  |  |  |  |
| General distress | Total | -.15 (.05) | -0.24, -0.06 | -3.37 | <.001 |  |
|  | Direct | -.13 (.04) | -0.21, -0.04 | -2.92 | .003 |  |
|  | Indirect | -.02 (.01) | -0.05, 0.001 | - | - | N/A (N/A) |
|  |  |  |  |  |  |  |
| Self-esteem | Total | .44 (.14) | 0.17, 0.71 | 3.20 | .002 |  |
|  | Direct | .40 (.14) | 0.13, 0.67 | 2.90 | .004 |  |
|  | Indirect | .04 (.03) | 0.004, 0.12 | - | - | .02 (-.03) |

*Note.* Sleep schedule variability is the mediator variable for all models. All Models have Dfs of 2, 373. N/A was reported for the effect size when there was no significant mediation effect (or reverse effect). See the Table 1 note in the main paper for further details.

Table S19
*The Mediating Effect of Sleep Disturbances on the Relationship Between Social Class and Each Outcome Variable (Study 3)*

| Outcome variables | Effect type | *b* (*SE*) | 95% CIs | *t* | *P* | CSIES (Reverse) |
| --- | --- | --- | --- | --- | --- | --- |
| Physical health | Total | -.30 (.08) | -0.45, -0.15 | -3.87 | <.001 |  |
| Symptoms | Direct | -.16 (.07) | -0.30, -0.03 | -2.43 | .016 |  |
|  | Indirect | -.13 (.04) | -0.22, -0.06 | - | - | -.08 (-.09) |
|  |  |  |  |  |  |  |
| General physical | Total | -.33 (.08) | -0.50, -0.17 | -3.97 | <.001 |  |
| Health | Direct | -.23 (.08) | -0.39, -0.08 | -2.92 | .004 |  |
|  | Indirect | -.10 (.03) | -0.17, -0.04 | - | - | -.06 (-.06) |
|  |  |  |  |  |  |  |
| General distress | Total | -.21 (.04) | -0.29, -0.13 | -5.01 | <.001 |  |
|  | Direct | -.13 (.04) | -0.20, -0.06 | -3.66 | <.001 |  |
|  | Indirect | -.08 (.03) | -0.13, -0.03 | - | - | -.09 (-.12) |
|  |  |  |  |  |  |  |
| Self-esteem | Total | .46 (.12) | 0.22, 0.69 | 3.73 | <.001 |  |
|  | Direct | .31 (.12) | 0.08, 0.54 | 2.64 | .001 |  |
|  | Indirect | .15 (.05) | 0.06, 0.25 | - | - | .06 (-.06) |

*Note.* Sleep disturbances is the mediator variable for all models. All Models have Dfs of 2, 443. See the Table 1 note in the main paper for further details.

Table S20
*The Mediating Effect of Sleep Duration on the Relationship Between Social Class and Each Outcome Variable (Study 3)*

| Outcome variables | Effect type | *b* (*SE*) | 95% CIs | *t* | *p* | CSIES (Reverse) |
| --- | --- | --- | --- | --- | --- | --- |
| Physical health | Total | -.30 (.08) | -0.45, -0.15 | -3.90 | <.001 |  |
| Symptoms | Direct | -.28 (.08) | -0.44, -0.13 | -3.67 | <.001 |  |
|  | Indirect | -.02 (.01) | -0.05, 0.002 | - | - | N/A (N/A) |
|  |  |  |  |  |  |  |
| General physical | Total | -.33 (.08) | -0.50, -0.17 | -3.97 | <.001 |  |
| Health | Direct | -.30 (.08) | -0.46, -0.14 | -3.60 | <.001 |  |
|  | Indirect | -.03 (.02) | -0.07, -0.004 | - | - | -.02 (-.03) |
|  |  |  |  |  |  |  |
| General distress | Total | -.21 (.04) | -0.30, -0.13 | -5.01 | <.001 |  |
|  | Direct | -.19 (.04) | -0.27, -0.11 | -4.58 | <.001 |  |
|  | Indirect | -.02 (.01) | -0.04, -0.003 | - | - | -.02 (-.05) |
|  |  |  |  |  |  |  |
| Self-esteem | Total | .46 (.12) | 0.22, 0.70 | 3.80 | <.001 |  |
|  | Direct | .43 (.12) | 0.19, 0.67 | 3.55 | <.001 |  |
|  | Indirect | .03 (.02) | -0.001, 0.07 | - | - | N/A (N/A) |

*Note.* Sleep duration is the mediator variable for all models. All Models have Dfs of 2, 443. N/A was reported for the effect size when there was no significant mediation effect (or reverse effect). See the Table 1 note in the main paper for further details.

Table S21
*The Mediating Effect of Presleep Worries on the Relationship Between Social Class and Each Outcome Variable (Study 3)*

| Outcome variables | Effect type | *b* (*SE*) | 95% CIs | *t* | *P* | CSIES (Reverse) |
| --- | --- | --- | --- | --- | --- | --- |
| Physical health | Total | -.30 (.08) | -0.45, -0.15 | -3.87 | <.001 |  |
| symptoms | Direct | -.18 (.07) | -0.32, 0.05 | -2.64 | .001 |  |
|  | Indirect | -.12 (.04) | -0.19, -0.04 | - | - | -.07 (-.08) |
|  |  |  |  |  |  |  |
| General physical | Total | -.33 (.08) | -0.50, -0.17 | -3.97 | <.001 |  |
| health | Direct | -.23 (.08) | -0.39, -0.08 | -2.95 | .003 |  |
|  | Indirect | -.10 (.03) | -0.16, -0.04 | - | - | -.06 (-.07) |
|  |  |  |  |  |  |  |
| General distress | Total | -.21 (.04) | -0.29, -0.13 | -5.01 | <.001 |  |
|  | Direct | -.14 (.04) | -0.21, -0.07 | -3.81 | <.001 |  |
|  | Indirect | -.07 (.02) | -0.12, -0.03 | - | - | -.08 (-.12) |
|  |  |  |  |  |  |  |
| Self-esteem | Total | .46 (.12) | 0.22, 0.69 | 3.73 | <.001 |  |
|  | Direct | .30 (.11) | 0.08, 0.52 | 2.63 | .001 |  |
|  | Indirect | .16 (.05) | 0.06, 0.25 | - | - | .06 (-.07) |

*Note.* Presleep worries is the mediator variable for all models. All Models have Dfs of 2, 443. See the Table 1 note in the main paper for further details.

## Detailed Comparison of Sleep Measures as Mediators

The results of comparing the sleep measures simultaneously in mediation analyses are detailed in this section. Within Tables S22-24, the CSIES for each sleep mediator when included in a mediation model between social class and one of the health variables is shown. These results demonstrate which sleep mediators remained significant, and which became non-significant (implying shared variance between sleep mediators). The final column of each table provides an average absolute effect size, which provides an average effect size of the sleep mediator across each mediation model. Across all studies, presleep worries is shown to have the highest average absolute effect size, suggesting that, compared to the other components, this component of sleep is more relevant and useful to consider in the relation between social class and health.

Table S22
*Comparison of Indirect Effect Sizes (CSIES) Between Sleep Components for Each Outcome Variable (Study 1)*

|  | Outcome variables | | | | |
| --- | --- | --- | --- | --- | --- |
|  | Physical health symptoms | General physical health | General distress | Self-esteem | Average absolute effect size |
| Presleep worries | -.028^*^ | -.025^*^ | -.048^*^ | .032^*^ | .033 |
| Sleep disturbances | -.034^*^ | -.016^*^ | -.022^*^ | .006 | .020 |
| Sleep duration | -.004 | -.016^*^ | -.007 | .009^*^ | .009 |
| Sleep schedule variability | -.004 | -.009^*^ | -.001 | .006 | .005 |

*Note*. The average absolute effect sizes are ordered from highest absolute average effect size to lowest.

* = significant mediation effects at *p* < .05.

Table S23
*Comparison of Indirect Effect Sizes (CSIES) Between Sleep Components for Each Outcome Variable (Study 2)*

|  | Outcome variables | | | | |
| --- | --- | --- | --- | --- | --- |
|  | Physical health symptoms | General physical health | General distress | Self-esteem | Average absolute effect size |
| Presleep worries | -.052^*^ | -.030^*^ | -.060^*^ | .037^*^ | .045 |
| Sleep schedule variability | -.004 | -.018^*^ | -.015 | .010 | .012 |
| Sleep duration | -.007 | -.002 | -.013^*^ | - | .007 |

*Note*. The average absolute effect sizes are ordered from highest absolute average effect size to lowest. Sleep disturbances was not included because it was not correlated with social class. Similarly, sleep duration was not included in the test with self-esteem because it was not correlated with self-esteem.

* = significant mediation effects at *p* < .05.

Table S24
*Comparison of Indirect Effect Sizes (CSIES) Between Sleep Components for Each Outcome Variable (Study 3)*

|  | Outcome variables | | | | |
| --- | --- | --- | --- | --- | --- |
|  | Physical health symptoms | General physical health | General distress | Self-esteem | Average absolute effect size |
| Presleep worries | -.037^*^ | -.039^*^ | -.048^*^ | .047^*^ | .043 |
| Sleep disturbances | -.058^*^ | -.019 | -.047^*^ | .019 | .036 |
| Sleep duration | -.009 | -.004 | -.002 | -.005 | .005 |
| Sleep schedule variability | -.0001 | -.001 | -.001 | .001 | .001 |

*Note*. The average absolute effect sizes are ordered from highest absolute average effect size to lowest.

* = significant mediation effects at *p* < .05.

## Detailed Correlations for Hypothesis 2

Table S25
*Perceived Feasibility of Sleep Hygiene Techniques: Means, Standard Deviations, Cronbach Alpha and Correlations Coefficients With Social Class (Study 3)*

|  | *M* | | *SD* | | Cronbach alpha | | Relationship with social class | |
| --- | --- | --- | --- | --- | --- | --- | --- | --- |
| Promoting good sleep subscale | 3.56 | 0.82 | | 0.78 | | .06 | |  |
| Sleep environment subscale | 4.38 | 0.90 | | 0.62 | | .12^**^ | |  |

*Note*. The response scales the sleep hygiene feasibility subscales ranged from 0 to 6.

***p* < .01.

Table S26
*Correlations Between Perceived Feasibility of Sleep Hygiene Techniques Subscales and Sleep Measures (Study 3)*

|  | Promoting good sleep subscale | Sleep environment subscale |
| --- | --- | --- |
| Sleep quality | -.31^**^ | -.31^**^ |
| Daytime sleepiness | -.23^**^ | -.19^**^ |
| Sleep disturbances | -.31^**^ | -.33^**^ |
| Sleep duration | -.15^**^ | -.17^**^ |
| Presleep worries | -.21^**^ | -.25^**^ |
| Sleep schedule variability | -.45^**^ | -.15^**^ |

***p* < .01.

## Sleep Hygiene Serial Mediation

Our exploratory analyses also tested the role of perceived feasibility of sleep environment hygiene in the social class 🡪 sleep 🡪 health mediation model (Model 6 in PROCESS). We added sleep hygiene immediately after social class to create a serial mediation test as follows: social class 🡪 perceived feasibility of sleep environment hygiene 🡪 sleep 🡪 health. The results indicated significant serial mediation effects, with only one test failing to demonstrate a significant serial mediation effect (social class 🡪 perceived feasibility of sleep environment hygiene 🡪 sleep duration 🡪 anxiety).

References

Blais, A.-R., & Weber, E. U. (2006). A Domain-specific Risk-taking (DOSPERT) Scale for adult populations. *Judgment and Decision Making*, *1*, 33-47. https://doi.org/2007-04381-004

Carver, C. S. (1997). You want to measure coping but your protocol’s too long: Consider the Brief COPE. *International Journal of Behavioral Medicine*, *4*, 92-100. https://doi.org/10.1207/s15327558ijbm0401_6

Cutrona, C. E., & Russell, D. W. (1987). The provisions of social relationships and adaptation to stress. *Advances in Personal Relationships*, *1*, 37-67.

Darmon, N., & Drewnowski, A. (2008). Does social class predict diet quality? *The American Journal of Clinical Nutrition*, *87*, 1107-1117. https://doi.org/1093/ajcn/87.5.1107

Eisinga, R., Te Grotenhuis, M., & Pelzer, B. (2013). The reliability of a two-item scale: Pearson, Cronbach, or Spearman-Brown? *International Journal of Public Health*, *58*, 637-642. https://doi.org/10.1007/s00038-012-0416-3

Florian, V., & Mikulincer, M. (1997). Fear of personal death in adulthood: The impact of early and recent losses. *Death Studies*, *21*(1), 1-24. https://doi.org/10.1080/074811897202119

Hurley, K. M., Oberlander, S. E., Merry, B. C., Wrobleski, M. M., Klassen, A. C., & Black, M. M. (2009). The healthy eating index and youth healthy eating index are unique, nonredundant measures of diet quality among low-income, African American adolescents. *The Journal of Nutrition*, *139*, 359-364. https://doi.org/10.3945/jn.108.097113

Jarvis, M. J. (1993). Does caffeine intake enhance absolute levels of cognitive performance? *Psychopharmacology*, *110*, 45-52. https://doi.org/10.1007/BF02246949

Johnson, S. L., Solomon, B. S., Shields, W. C., McDonald, E. M., McKenzie, L. B., & Gielen, A. C. (2009). Neighborhood violence and its association with mothers’ health: assessing the relative importance of perceived safety and exposure to violence. *Journal of Urban Health*, *86*, 538-550. https://doi.org/10.1007/s11524-009-9345-8

Leach, C. W., van Zomeren, M., Zebel, S., Vliek, M. L. W., Pennekamp, S. F., Doosje, B., ... Spears, R. (2008). Group-level self-definition and self-investment: A hierarchical (multicomponent) model of in-group identification. *Journal of Personality and Social Psychology*, *95*, 144-165. https://doi.org/10.1037/0022-3514.95.1.144

Nielsen, T. A., Stenstrom, P., & Levin, R. (2006). Nightmare frequency as a function of age, gender, and September 11, 2001: Findings from an Internet questionnaire. *Dreaming*, *16*, 145-158. https://doi.org/10.1037/1053-0797.16.3.145

Nielsen, T. A., Zadra, A. L., Simard, V., Saucier, S., Stenstorm, P., Smith, C., & Kuiken, D. (2003). The typical dreams of Canadian university students. Dreaming, 13, 211-235.

Rubin M., Evans O., & Wilkinson, R. B. (2016). A longitudinal study of the relations between university students’ subjective social status, social contact with university friends, and mental health and well-being. *Journal of Social and Clinical Psychology*, *35*, 722-737. https://doi.org/10.1521/jscp.2016.35.9.722

Rubin, M., & Kelly, B. M. (2015). A cross-sectional investigation of parenting style and friendship as mediators of the relation between social class and mental health in a university community. *International Journal for Equity in Health*, *14*(87), 1-11. https://doi.org/10.1186/s12939-015-0227-2

Rubin, M., & Wright, C. L. (2015b). Time and money explain social class differences in students’ social integration at university. *Studies in Higher Education*. https://doi.org/10.1080/03075079.2015.1045481

Rubin, M., & Stuart, R. (2017). Kill or cure? Different types of social identification amplify and buffer the relation between social class and mental health. Unpublished manuscript. The University of Newcastle, Australia.

Sampson, R. J., Raudenbush, S. W., & Earls, F. (1997). Neighborhoods and violent crime: A multilevel study of collective efficacy. *Science*, *277*, 918-924.

Schwarzer, R., & Jerusalem, M. (1995). Generalized Self-Efficacy scale. In J. Weinman, S. Wright, & M. Johnston, *Measures in health psychology*: A user’s portfolio. Causal and control beliefs (pp. 35-37). NFER-NELSON.

Terrill, A. L., Gjerde, J. M., & Garofalo, J. P. (2015). Background Stress Inventory: Developing a measure of understudied stress. *Stress and Health*, *31*, 290-298. https://doi.org/10.1002/smi.2554

Zadra, A., & Donderi, D. C. (2000). Nightmares and bad dreams: their prevalence and relationship to well-being. *Journal of Abnormal Psychology*, *109*, 273-281. https://doi.org/10.1037/0021-843X.109.2.273

Zadra, A., Pilon, M., & Donderi, D. C. (2006). Variety and intensity of emotions in nightmares and bad dreams. *The Journal of Nervous and Mental Disease*, *194*, 249-254. https://doi.org/10.1097/01.nmd.0000207359.46223.dc
